# Parsimonious hidden Markov models for matrix-variate longitudinal data

**DOI:** 10.1007/s11222-022-10107-0

**Published:** 2022-06-15

**Authors:** Salvatore D. Tomarchio, Antonio Punzo, Antonello Maruotti

**Affiliations:** 1 Dipartimento di Economia e Impresa, Università degli Studi di Catania, Catania, Italia; 2 Dipartimento di Giurisprudenza, Economia, Politica e Lingue Moderne, Libera Università Maria Ss. Assunta, Roma, Italia; 3 Department of Mathematics, University of Bergen, Bergen, Norway

**Keywords:** Hidden Markov models, Matrix-variate, Clustering, Parsimonious models

## Abstract

Hidden Markov models (HMMs) have been extensively used in the univariate and multivariate literature. However, there has been an increased interest in the analysis of matrix-variate data over the recent years. In this manuscript we introduce HMMs for matrix-variate balanced longitudinal data, by assuming a matrix normal distribution in each hidden state. Such data are arranged in a four-way array. To address for possible overparameterization issues, we consider the eigen decomposition of the covariance matrices, leading to a total of 98 HMMs. An expectation-conditional maximization algorithm is discussed for parameter estimation. The proposed models are firstly investigated on simulated data, in terms of parameter recovery, computational times and model selection. Then, they are fitted to a four-way real data set concerning the unemployment rates of the Italian provinces, evaluated by gender and age classes, over the last 16 years.

## Introduction

Multivariate longitudinal data have been widely analyzed in the literature (Verbeke et al. 2014 and Verdam and Oort 2019). By focusing on balanced data, i.e. those where each unit is observed in all times, they are usually presented in the standard three-way format, where units, times and variables are arranged in software-ready manners. Because of their three-way structure, multivariate balanced longitudinal data have been recently arranged in a matrix-variate fashion (Huang et al. 2019 and Viroli 2011b): for each unit $i=1,\dots ,I$, we observe a $P \times T$ matrix, where *P* and *T* denote the number of variables and times, respectively. Then, such data have been used for model-based clustering via matrix-variate mixture models (see e.g. Melnykov and Zhu 2019; Tomarchio et al. 2022, 2020 and Zhu and Melnykov 2021). This allows for both clustering units in homogeneous groups, defined according to similarities between matrix-variate data, and separately modeling the association between variables and times. Unfortunately, this procedure has two side effects:

- using the time on either the rows or the columns of the matrices reduces the types of longitudinal data structures that can be arranged in a matrix-variate framework. For instance, spatio-temporal data are used either to analyze *P* variables observed at *T* times for *R* different locations (Viroli 2011b) or to evaluate one measurement on *R* locations at *T* times on a set of *I* units (Viroli 2011a). However, it is not possible to jointly consider *P* variables at *R* locations for *T* times on *I* units. A possible solution could be to combine locations-times in a single *RT*-dimension, as done by Viroli (2011a), but this implies a loss in terms of interpretability as well as an increase in the number of parameters of the estimated models, given the higher dimensionality of the matrices.
  Another example consists of two-factor data, which have been commonly considered in longitudinal settings (see e.g. Brunner and Puri 2001; Fitzmaurice and Ravichandran 2008; Noguchi et al. 2012). Such data have been recently used in matrix-variate mixture models by Sarkar et al. (2020) in a not-longitudinal way, given that the factors fill the two dimensions of the $P \times R$ matrices for the *I* units, and an additional dimension for the time is required.
  To summarize, it would be necessary to move from three-way to four-way arrays in order to properly consider and model all the discussed data features.
- The matrix-variate clustering approaches mentioned in (a) assume time-constant clustering, i.e. it is not possible for the sample units to move across clusters over time and the evolution over time of the clustering structure is completely overlooked. Time-varying heterogeneity is a specific important feature of longitudinal data analysis and, as such, appropriate modeling strategies should be considered. Hidden Markov models (HMMs) have been extensively used to address this longitudinal data peculiarity (Altman 2007; Maruotti 2011; Bartolucci et al. 2012; Zucchini et al. 2017). Being (dependent) mixtures, HMMs simultaneously allow for clustering units and for modeling the evolution of the clustering over time.

To jointly consider the aspects in (a) and (b), in this manuscript we introduce and discuss HMMs for matrix-variate balanced longitudinal data (MV-HMMs), with a specific application on the two-factor longitudinal case. Such kind of data can be arranged in a four-way array of dimension $P \times R \times I \times T$. A side effect of working with four-way data is the potentially large number of parameters involved. This often occurs because of the (row- and column-specific) covariance matrices, since $P(P+1)/2$ and $R(R+1)/2$ unique parameters must be estimated. One of the most classical ways of addressing this overparameterization issue involves the eigen decomposition of the covariance matrices introduced by Celeux and Govaert (1995). This decomposition offers remarkable flexibility and a geometric interpretation in terms of volume, shape and orientation of the hidden states (for other approaches available in the HMMs literature, see Maruotti et al. 2017 and Farcomeni et al. 2020). By using the eigen decomposition of the covariance matrices, we obtain a family of 98 parsimonious MV-HMMs that will be described in Sect. 2.2, after the presentation of the general model (Sect. 2.1). In this framework, model parameters can be estimated by a full maximum likelihood method based on the Expectation Conditional Maximization (ECM) algorithm (Meng and Rubin 1993), and recursions widely used in the HMM literature (Baum et al. 1970). An iterative Minorization-Maximization (MM) algorithm (Browne and McNicholas 2014) is also adopted to update some of the parameters related to a subset of the parsimonious MV-HMMs, during the ECM algorithm.

In Sect. 3, we illustrate the proposal by a large-scale simulation study in order to investigate the empirical behavior of the proposed approach with respect to several aspects, such as the number of observed times, the number of hidden states, the data dimensionality and the association structure between factor-levels. We focus on goodness of clustering and parameters recovery, with a focus on computational times and model selection procedures. Furthermore, in Sect. 4 we test the proposal by analyzing a sample taken from the Italian National Institute of Statistics on the unemployment rate in 98 Italian provinces recorded for 16 years, also covering the 2008 crisis. We examine the unemployment rate arranged as a two-factor design, i.e. taking into account gender and age classes, by allowing some dynamics in the evolution of unemployment. We obtain a flexible model by including different associations across levels, changing according to the inferred dynamics, and by accounting for unobserved characteristics influencing changes in the province’s unemployment patterns. For comparison purposes, we added two competing approaches that could be used if our models were not available, thus coercing the data in a three-way structure: (i) mixtures of parsimonious matrix-variate normal distributions and (ii) parsimonious multivariate normal HMMs. Finally, Sect. 5 summarizes the key aspects of our proposal along with future possible extensions.

## Methodology

### The model

Let $\left\{ {\mathcal {X}}_{it}; i=1,\ldots ,I, t=1,\ldots ,T\right\}$ be a sequence of matrix-variate balanced longitudinal observations recorded on *I* units over *T* times, with ${\mathcal {X}}_{it}\in {\mathbb {R}}^{P\times R}$, and let $\left\{ S_{it}; i=1,\ldots ,I, t=1,\ldots ,T\right\}$ be a first-order Markov chain defined on the state space $\left\{ 1,\ldots ,k,\ldots ,K\right\}$. As mentioned in Sect. 1, a HMM is a particular type of dependent mixture model consisting of two parts: the underlying unobserved process $\left\{ S_{it}\right\}$ that satisfies the Markov property, i.e.

$$\begin{aligned}&\text {Pr}\left( S_{it}=s_{it} | S_{i1}=s_{i1}, \ldots ,S_{it-1}=s_{it-1}\right) \\&\quad =\text {Pr}\left( S_{it}=s_{it} | S_{it-1}=s_{it-1}\right) , \end{aligned}$$

and the state-dependent observation process $\left\{ {\mathcal {X}}_{it}\right\}$ for which the conditional independence property holds, i.e.

$$\begin{aligned} f\Big ({\mathcal {X}}_{it}&={\mathbf {X}}_{it} | {\mathcal {X}}_{i1}={\mathbf {X}}_{i1}, \ldots ,{\mathcal {X}}_{it-1}={\mathbf {X}}_{it-1},S_{i1}=\\&=s_{i1}\ldots ,S_{it}=s_{it}\Bigg ) = f\left( {\mathcal {X}}_{it}={\mathbf {X}}_{it} | S_{it}=s_{it} \right) , \end{aligned}$$

where $f(\cdot )$ is a generic probability density function (pdf). Therefore, the unknown parameters in an HMM involve both the parameters of the Markov chain and those of the state-dependent pdfs. In detail, the parameters of the Markov chain are the initial probabilities $\pi _{ik}=\text {Pr}\left( S_{i1}=k\right)$, $k=1,\ldots ,K$, being *K* the number of states, and the transition probabilities

$$\begin{aligned} \pi _{ik|j}= & {} \text {Pr}\left( S_{it}=k|S_{it-1}=j\right) , t=2,\ldots ,T \text {and} \\&\quad j,k=1,\ldots ,K, \end{aligned}$$

where *k* refers to the current state and *j* refers to the one previously visited. To simplify the discussion, we will consider homogeneous HMMs, that is $\pi _{ik|j}=\pi _{k|j}$ and $\pi _{ik}=\pi _{k}, i=1,\ldots ,I$. We collect the initial probabilities in the *K*-dimensional vector $\varvec{\pi }$, whereas the time-homogenous transition probabilities are inserted in the $K \times K$ transition matrix $\varvec{\Pi }$.

**Table 1:** Nomenclature, covariance matrix structure, and number of free parameters in $\varvec{\Phi }_{1},\ldots ,\varvec{\Phi }_{K}$ for the parsimonious models obtained via the eigen decomposition of the state covariance matrices. ${\varvec{I}}$ is the identity matrix

Family	Model	Type	Volume	Shape	Orientation	# of free parameters in $\varvec{\Phi }_{1},\ldots ,\varvec{\Phi }_{K}$
Spherical	EII	$\lambda {\varvec{I}}$	Equal	Spherical	–	1
Spherical	VII	$\lambda _{k}{\varvec{I}}$	Variable	Spherical	–	*K*
Diagonal	EEI	$\lambda \varvec{\Delta }$	Equal	Equal	Axis-Aligned	*Q*
Diagonal	VEI	$\lambda _{k}\varvec{\Delta }$	Variable	Equal	Axis-Aligned	$K + Q - 1$
Diagonal	EVI	$\lambda \varvec{\Delta }_{k}$	Equal	Variable	Axis-Aligned	$K(Q-1)+1$
Diagonal	VVI	$\lambda _{k}\varvec{\Delta }_{k}$	Variable	Variable	Axis-Aligned	*KQ*
General	EEE	$\lambda \varvec{\Gamma }\varvec{\Delta }\varvec{\Gamma }'$	Equal	Equal	Equal	$Q(Q+1)/2$
General	VEE	$\lambda _{k}\varvec{\Gamma }\varvec{\Delta }\varvec{\Gamma }'$	Variable	Equal	Equal	$Q(Q+1)/2 + K - 1$
General	EVE	$$\lambda \varvec{\Gamma }\varvec{\Delta }_{k}\varvec{\Gamma }'$$	Equal	Variable	Equal	$$Q(Q-1)/2 + K(Q-1)+1 $$
General	VVE	$\lambda _{k}\varvec{\Gamma }\varvec{\Delta }_{k}\varvec{\Gamma }'$	Variable	Variable	Equal	$Q(Q-1)/2 + KQ$
General	EEV	$\lambda \varvec{\Gamma }_{k}\varvec{\Delta }\varvec{\Gamma }_{k}'$	Equal	Equal	Variable	$KQ(Q-1)/2 + Q$
General	VEV	$\lambda _{k}\varvec{\Gamma }_{k}\varvec{\Delta }\varvec{\Gamma }_{k}'$	Variable	Equal	Variable	$KQ(Q-1)/2 + K + Q - 1$
General	EVV	$\lambda \varvec{\Gamma }_{k}\varvec{\Delta }_{k}\varvec{\Gamma }_{k}'$	Equal	Variable	Variable	$KQ(Q+1)/2 -K +1$
General	VVV	$\lambda _{k}\varvec{\Gamma }_{k}\varvec{\Delta }_{k}\varvec{\Gamma }_{k}'$	Variable	Variable	Variable	$KQ(Q+1)/2$

Regarding the conditional density for the observed process, it will be given by a matrix-normal distribution, i.e.

1 $$\begin{aligned}&\phi \left( {\mathbf {X}}_{it}|S_{it}=k;\varvec{\theta }_{k}\right) \nonumber \\&\quad = \frac{\exp \left\{ -\frac{1}{2}\,\text{ tr }\left[ \varvec{\Sigma }_{k}^{-1}({\mathbf {X}}-{\mathbf {M}}_{k})\varvec{\Psi }_{k}^{-1}({\mathbf {X}}-{\mathbf {M}}_{k})'\right] \right\} }{(2\pi )^{\frac{PR}{2}}|\varvec{\Sigma }_{k}|^{\frac{R}{2}}|\varvec{\Psi }_{k}|^{\frac{P}{2}}}, \end{aligned}$$

where ${\mathbf {M}}_{k}$ is the $P \times R$ matrix of means, $\varvec{\Sigma }_{k}$ is the $P\times P$ covariance matrix containing the covariances between the P rows, $\varvec{\Psi }_{k}$ is the $R\times R$ covariance matrix containing the covariances of the *R* columns and $\varvec{\theta }_{k}=\left\{ {\mathbf {M}}_{k},\varvec{\Sigma }_{k},\varvec{\Psi }_{k}\right\}$. For an exhaustive description of the matrix-normal distribution and its properties see Gupta and Nagar (2018).

### Parsimonious models

As discussed in Sect. 1, a way to reduce the number of parameters of the model is to introduce parsimony in the covariance matrices via the well-known eigen decomposition introduced by Celeux and Govaert (1995). Specifically, a $Q \times Q$ covariance matrix can be decomposed as

2 $$\begin{aligned} \varvec{\Phi }_{k} = \lambda _{k}\varvec{\Gamma }_{k}\varvec{\Delta }_{k}\varvec{\Gamma }_{k}', \end{aligned}$$

where $\lambda _{k}=|\varvec{\Phi }_{k}|^{1/Q}$, $\varvec{\Gamma }_{k}$ is a $Q \times Q$ orthogonal matrix of the eigenvectors of $\varvec{\Phi }_{k}$ and $\varvec{\Delta }_{k}$ is the $Q \times Q$ diagonal matrix with the scaled eigenvalues of $\varvec{\Phi }_{k}$ (such that $|\varvec{\Delta }_{k}| = 1$) located on the main diagonal. The decomposition in (2) has some useful practical interpretations. From a geometric point of view, $\lambda _{k}$ determines the volume, $\varvec{\Gamma }_{k}$ governs the orientation, and $\varvec{\Delta }_{k}$ denotes the shape of the *k*th state. From a statistical point of view, as well-documented in Greselin and Punzo (2013), Bagnato and Punzo (2021) and Punzo and Bagnato (2021), the columns of $\varvec{\Gamma }_{k}$ govern the orientation of the principal components (PCs) of the *k*th state, the diagonal elements in $\varvec{\Delta }_{k}$ are the normalized variances of these PCs, and $\lambda _{k}$ can be meant as the overall volume of the scatter in the space spanned by the PCs of the *k*th state. By imposing constraints on the three components of (2), the fourteen parsimonious models of Table 1 are obtained.

Considering that we have two covariance matrices in (1), this would yield to $14 \times 14 = 196$ parsimonious MV-HMMs. However, there is a non-identifiability issue since $\varvec{\Psi }\otimes \varvec{\Sigma }= \varvec{\Psi }^{*} \otimes \varvec{\Sigma }^{*}$ if $\varvec{\Sigma }^{*}= a\varvec{\Sigma }$ and $\varvec{\Psi }^{*}= a^{-1}\varvec{\Psi }$. As a result, $\varvec{\Sigma }$ and $\varvec{\Psi }$ are identifiable up to a multiplicative constant *a* (Sarkar et al. 2020). To avoid such issue, the column covariance matrix $\varvec{\Psi }$ is restricted to have $|\varvec{\Psi }|= 1$, implying that in (2) the parameter $\lambda _{k}$ is unnecessary. This reduces the number of models related to $\varvec{\Psi }$ from 14 to 7, i.e., ${\varvec{I}}, \varvec{\Delta }, \varvec{\Delta }_{k}, \varvec{\Gamma }\varvec{\Delta }\varvec{\Gamma }',\varvec{\Gamma }\varvec{\Delta }_{k}\varvec{\Gamma }', \varvec{\Gamma }_{k}\varvec{\Delta }\varvec{\Gamma }_{k}', \varvec{\Gamma }_{k}\varvec{\Delta }_{k}\varvec{\Gamma }_{k}'$. Therefore, we obtain $14 \times 7 = 98$ parsimonious MV-HMMs.

### Maximum likelihood estimation

To fit our MV-HMMs, we use the expectation-conditional maximization (ECM) algorithm (Meng and Rubin 1993). The ECM algorithm is a variant of the classical expectation-maximization (EM) algorithm (Dempster et al. 1977), from which it differs since the M-step is replaced by a sequence of simpler and computationally convenient CM-steps.

Let ${\mathcal {S}}=\left\{ {\mathbf {X}}_{it}; i=1,\ldots ,I, t=1,\ldots ,T\right\}$ be a sample of matrix-variate balanced longitudinal observations. Then, the incomplete-data likelihood function is

$$\begin{aligned} L\left( \varvec{\Theta }|S\right)= & {} \prod _{i=1}^{I} \varvec{\pi }' \varvec{\phi }\left( {\mathbf {X}}_{i1}\right) \varvec{\Pi }\varvec{\phi }\left( {\mathbf {X}}_{i2}\right) \varvec{\Pi }\ldots \varvec{\phi }\left( {\mathbf {X}}_{iT-1}\right) \\&\quad \varvec{\Pi }\varvec{\phi }\left( {\mathbf {X}}_{iT}\right) {\varvec{1}}_K, \end{aligned}$$

where $\varvec{\phi }\left( {\mathbf {X}}_{it}\right)$ is a $K \times K$ diagonal matrix with conditional densities $\phi \left( {\mathcal {X}}_{it}={\mathbf {X}}_{it} | S_{it}=k\right)$ on the main diagonal, ${\varvec{1}}_K$ is a vector *K* ones and $\varvec{\Theta }$ contains all the model parameters. In this setting, ${\mathcal {S}}$ is viewed as incomplete because, for each observation, we do not know its state membership and its evolution over time. For this reason, let us define the unobserved state membership ${\varvec{z}}_{it}=\left( z_{it1},\ldots ,z_{itk},\ldots ,z_{itK}\right) '$ and the unobserved states transition

$$\begin{aligned}{\varvec{z}}{\varvec{z}}_{it}= \begin{bmatrix} zz_{it11} &{} \ldots &{} zz_{it1k} &{} \ldots &{} zz_{it1K}\\ \vdots &{} &{} \vdots &{} &{} \vdots \\ zz_{itj1} &{} \ldots &{} zz_{itjk} &{} \ldots &{} zz_{itjK}\\ \vdots &{} &{} \vdots &{} &{} \vdots \\ zz_{itK1} &{} \ldots &{} zz_{itKk} &{} \ldots &{} zz_{itKK}\end{bmatrix}, \end{aligned}$$

where

$$\begin{aligned} z_{itk}= & {} {\left\{ \begin{array}{ll} 1 &{} \quad \text {if} S_{it}=k \\ 0 &{} \quad \text {otherwise} \end{array}\right. } \quad \text {and}\\ zz_{itjk}= & {} {\left\{ \begin{array}{ll} 1 &{} \quad \text {if} S_{it-1}=j \text {and} S_{it}=k \\ 0 &{} \quad \text {otherwise} \end{array}\right. }. \end{aligned}$$

Therefore, the complete data are ${\mathcal {S}}_c=\Big \{{\mathbf {X}}_{it},{\varvec{z}}_{it},{\varvec{z}}{\varvec{z}}_{it}; i=1,\ldots ,I, t=1,\ldots ,T\Big \}$ and the corresponding complete-data log-likelihood is

3 $$\begin{aligned} l_{c}\left( \varvec{\Theta }|{\mathcal {S}}_{c}\right) = l_{c_1}\left( \varvec{\pi }|{\mathcal {S}}_{c}\right) +l_{c_2}\left( \varvec{\Pi }|{\mathcal {S}}_{c}\right) +l_{c_3}\left( \varvec{\theta }|{\mathcal {S}}_{c}\right) , \end{aligned}$$

with $$\varvec{\theta }=\left\{ \varvec{\theta }_k; k=1,\ldots ,K\right\} $$ and

$$\begin{aligned} l_{c_1}\left( \varvec{\pi }|{\mathcal {S}}_{c}\right)&= \sum \limits _{i=1}^{I}\sum \limits _{k=1}^{K} z_{i1k} \log \left( \pi _k\right) \\ l_{c_2}\left( \varvec{\Pi }|{\mathcal {S}}_{c}\right)&= \sum \limits _{i=1}^{I}\sum \limits _{t=2}^{T}\sum \limits _{k=1}^{K}\sum \limits _{j=1}^{K} zz_{itjk} \log \left( \pi _{k|j}\right) \\ l_{c_3}\left( \varvec{\theta }|{\mathcal {S}}_{c}\right)&= \sum \limits _{i=1}^{I}\sum \limits _{t=1}^{T}\sum \limits _{k=1}^{K} z_{itk}\\&\quad \Bigg \{\Bigg .-\frac{PR}{2}\log \left( 2\pi \right) -\frac{R}{2}\log |\varvec{\Sigma }_{k}|-\frac{P}{2}\log |\varvec{\Psi }_{k}| \\&-\frac{1}{2}\,\text{ tr }\left[ \varvec{\Sigma }_{k}^{-1}({\mathbf {X}}_{it}-{\mathbf {M}}_{k})\varvec{\Psi }_{k}^{-1}({\mathbf {X}}_{it}-{\mathbf {M}}_{k})'\right] \Bigg . \Bigg \}. \end{aligned}$$

In the following, by adopting the notation used in Tomarchio et al. (2021a), the parameters marked with one dot will represent the updates at the previous iteration and those marked with two dots are the updates at the current iteration. Furthermore, we implemented the ECM algorithm used for fitting all the 98 parsimonious MV-HMMs in the HMM.fit() function of the **FourWayHMM** package (Tomarchio et al. 2021b) for the R statistical software (R Core Team 2019).

*E-Step* The E-step requires the calculation of the conditional expectation of (3), given ${\mathcal {S}}_{c}$ and the current estimates of ${\dot{\varvec{\Theta }}}$. Therefore, we need to replace $z_{itk}$ and $z_{itjk}$ with their conditional expectations, namely, $\ddot{z}_{itk}$ and $\ddot{zz}_{itjk}$. This can be efficiently done by exploiting a forward recursion approach (Baum et al. 1970; Baum 1972; Welch 2003).

Let us start by defining the forward probability

$$\begin{aligned} \gamma _{itk}=\text {Pr}\left( {\mathcal {X}}_{i1}={\mathbf {X}}_{i1},\ldots ,{\mathcal {X}}_{it}={\mathbf {X}}_{it},S_{it}=k\right) , \end{aligned}$$

that is the probability of seeing the partial sequence finishing up in state *k* at time *t*, and the corresponding backward probability

$$\begin{aligned} \beta _{itk}=\text {Pr}\left( {\mathcal {X}}_{it+1}={\mathbf {X}}_{it+1},\ldots ,{\mathcal {X}}_{iT}={\mathbf {X}}_{iT}|S_{it}=k\right) . \end{aligned}$$

It is known that the computation of the forward and backward probabilities is susceptible to numerical overflow errors (Farcomeni 2012). To prevent or at least to decrease the risk of such errors, the well known scaling procedure suggested by Durbin et al. (1998) can be implemented (for additional details, see also Zucchini et al. 2017). Then, the updates required in the E-step can be computed as

$$\begin{aligned} \ddot{z}_{itk}= & {} \frac{\gamma _{itk}\beta _{itk}}{\sum \limits _{h=1}^{K}\gamma _{ith}\beta _{ith}} \quad \text {and} \\ \ddot{zz}_{itjk}= & {} \frac{\gamma _{i\left( t-1\right) j}\pi _{k|j}\phi \left( {\mathbf {X}}_{it}|S_{it}=k\right) \beta _{itk}}{\sum \limits _{h=1}^{K}\gamma _{iTh}}. \end{aligned}$$

*CM-Step 1* Consider $\varvec{\Theta }=\left\{ \varvec{\Theta }_1,\varvec{\Theta }_2\right\}$, where $\varvec{\Theta }_1=\left\{ \pi _k,\varvec{\Pi },{\mathbf {M}}_{k},\varvec{\Sigma }_{k};k=1,\ldots ,K\right\}$ and $\varvec{\Theta }_2=\Big \{\varvec{\Psi }_{k};k=1,\ldots ,K\Big \}$. At the first CM-step, we maximize the expectation of (3) with respect to $\varvec{\Theta }_1$, fixing $\varvec{\Theta }_2$ at $\dot{\varvec{\Theta }_2}$. In particular, we obtain

$$\begin{aligned} \ddot{\pi }_k= & {} \frac{\sum _{i=1}^I \ddot{z}_{i1k}}{I}, \quad \ddot{\pi }_{k|j} = \frac{\sum _{i=1}^I \sum _{t=2}^T \ddot{zz}_{itjk}}{\sum _{i=1}^I \sum _{t=2}^T \sum _{k=1}^K \ddot{zz}_{itjk}} \quad \text {and} \\ \ddot{{\mathbf {M}}}_k= & {} \frac{\sum _{i=1}^I \sum _{t=1}^T \ddot{z}_{itk}{\mathbf {X}}_{it}}{\sum _{i=1}^I \sum _{t=1}^T \ddot{z}_{itk}}. \end{aligned}$$

The update for $\varvec{\Sigma }_k$ depends on the parsimonious structure considered. For notational simplicity, let ${\ddot{{\mathbf {Y}}}}=\sum _{k=1}^K {\ddot{{\mathbf {Y}}}}_k$ be the update of the within state row scatter matrix, where ${\ddot{{\mathbf {Y}}}}_k = \sum _{i=1}^I \sum _{t=1}^T \ddot{z}_{itk}\left( {\mathbf {X}}_{it}-{\ddot{{\mathbf {M}}}}_k\right) {{\dot{\varvec{\Psi }}}}_k^{-1}\left( {\mathbf {X}}_{it}-{\ddot{{\mathbf {M}}}}_k\right) '$ is the update of the row scatter matrix related to the *k*th state. The updates for the 14 parsimonious structures of $\varvec{\Sigma }_k$ are:

- Model EII [$\varvec{\Sigma }_k=\lambda {\varvec{I}}$]. In this setting, maximizing Eq. (3) reduces to the maximization of
  $$\begin{aligned} -\frac{PRTI}{2}\log \lambda -\frac{1}{2\lambda }\,\text{ tr }\left( {\ddot{{\mathbf {Y}}}}\right) . \end{aligned}$$
  Thus, we can obtain $\lambda$ as
  $$\begin{aligned} {\ddot{\lambda }} = \frac{\,\text{ tr }\left\{ {\ddot{{\mathbf {Y}}}}\right\} }{PRTI}. \end{aligned}$$
- Model VII [$\varvec{\Sigma }_k=\lambda _{k}{\varvec{I}}$]. In this case, maximizing Eq. (3) reduces to the maximization of
  $$\begin{aligned} -\frac{PR}{2} \sum \limits _{k=1}^{K} \log \lambda _k \sum \limits _{i=1}^{I}\sum \limits _{t=1}^{T} \ddot{z}_{itk}-\frac{1}{2}\sum \limits _{k=1}^{K}\frac{1}{\lambda _k}\,\text{ tr }\left( {\ddot{{\mathbf {Y}}}}_k\right) . \end{aligned}$$
  Thus, we can obtain $\lambda _k$ as
  $$\begin{aligned} {\ddot{\lambda }}_k = \frac{\,\text{ tr }\left\{ {\ddot{{\mathbf {Y}}}}_k\right\} }{PR \sum _{i=1}^I \sum _{t=1}^T \ddot{z}_{itk}}. \end{aligned}$$
- Model EEI [$\varvec{\Sigma }_k=\lambda \varvec{\Delta }$]. Here, maximizing Eq. (3) reduces to the maximization of
  $$\begin{aligned} -\frac{PRTI}{2}\log \lambda -\frac{1}{2\lambda }\,\text{ tr }\left( \varvec{\Delta }^{-1}{\ddot{{\mathbf {Y}}}}\right) . \end{aligned}$$
  Applying Corollary A.1 of Celeux and Govaert (1995), we can obtain $\lambda$ and $\varvec{\Delta }$ as
  $$\begin{aligned} {\ddot{\varvec{\Delta }}} =\frac{\text {diag}\left( {\ddot{{\mathbf {Y}}}}\right) }{\left| \text {diag}\left( {\ddot{{\mathbf {Y}}}}\right) \right| ^\frac{1}{P}} \quad \text {and} \quad {\ddot{\lambda }} = \frac{\left| \text {diag}\left( \ddot{{\mathbf {Y}}}\right) \right| ^\frac{1}{P}}{RTI}. \end{aligned}$$
- Model VEI [$\varvec{\Sigma }_k=\lambda _{k}\varvec{\Delta }$]. In this setting, maximizing Eq. (3) reduces to the maximization of
  $$\begin{aligned} -\frac{PR}{2}\sum \limits _{k=1}^{K} \log \lambda _k \sum \limits _{i=1}^{I}\sum \limits _{t=1}^{T} \ddot{z}_{itk}-\sum \limits _{k=1}^{K}\frac{1}{2\lambda _k}\,\text{ tr }\left( \varvec{\Delta }^{-1}{\ddot{{\mathbf {Y}}}}_k\right) . \end{aligned}$$
  Applying Corollary A.1 of Celeux and Govaert (1995), we can obtain $\varvec{\Delta }$ and $\lambda _{k}$ as
  $$\begin{aligned} {\ddot{\varvec{\Delta }}}= & {} \frac{\text {diag}\left( \sum \limits _{k=1}^K \dot{\lambda }_k^{-1}{\ddot{{\mathbf {Y}}}}_k\right) }{\left| \text {diag}\left( \sum \limits _{k=1}^K {{\dot{\lambda }}}_k^{-1}{\ddot{{\mathbf {Y}}}}_k\right) \right| ^\frac{1}{P}} \quad \text {and}\\ {\ddot{\lambda }}_k= & {} \frac{\,\text{ tr }\left\{ {\ddot{{\mathbf {Y}}}}_k {\ddot{\varvec{\Delta }}}^{-1}\right\} }{PR\sum _{i=1}^I \sum _{t=1}^T \ddot{z}_{itk}}. \end{aligned}$$
- Model EVI [$\varvec{\Sigma }_k=\lambda \varvec{\Delta }_{k}$]. In this case, maximizing Eq. (3) reduces to the maximization of
  $$\begin{aligned} -\frac{PRTI}{2}\log \lambda -\frac{1}{2\lambda }\sum \limits _{k=1}^{K} \,\text{ tr }\left( \varvec{\Delta }_k^{-1}{\ddot{{\mathbf {Y}}}}_k\right) . \end{aligned}$$
  Also in this case, by using Corollary A.1 of Celeux and Govaert (1995), we can obtain $\varvec{\Delta }_{k}$ and $\lambda$ as
  $$\begin{aligned} {\ddot{\varvec{\Delta }}}_k = \frac{\text {diag}\left( {\ddot{{\mathbf {Y}}}}_k\right) }{\left| \text {diag}\left( {\ddot{{\mathbf {Y}}}}_k\right) \right| ^\frac{1}{P}} \quad \text {and} \quad {\ddot{\lambda }} = \frac{\sum \limits _{k=1}^K\left| \text {diag}\left( {\ddot{{\mathbf {Y}}}}_k\right) \right| ^\frac{1}{P}}{RTI}. \end{aligned}$$
- Model VVI [$\varvec{\Sigma }_k=\lambda _{k}\varvec{\Delta }_{k}$]. Here, maximizing Eq. (3) reduces to the maximization of
  $$\begin{aligned} -\frac{PR}{2}\sum \limits _{k=1}^{K} \log \lambda _k \sum \limits _{i=1}^{I}\sum \limits _{t=1}^{T}\ddot{z}_{itk}-\sum \limits _{k=1}^{K}\frac{1}{2\lambda _k}\,\text{ tr }\left( \varvec{\Delta }_k^{-1}{\ddot{{\mathbf {Y}}}}_k\right) . \end{aligned}$$
  Again, by using Corollary A.1 of Celeux and Govaert (1995), we can obtain $\varvec{\Delta }_{k}$ and $\lambda _{k}$ as
  $$\begin{aligned} {\ddot{\varvec{\Delta }}}_k = \frac{\text {diag}\left( {\ddot{{\mathbf {Y}}}}_k\right) }{\left| \text {diag}\left( {\ddot{{\mathbf {Y}}}}_k\right) \right| ^\frac{1}{P}}\quad \text {and} \quad {\ddot{\lambda }}_k = \frac{\left| \text {diag}\left( {\ddot{{\mathbf {Y}}}}_k\right) \right| ^\frac{1}{P}}{R\sum _{i=1}^I \sum _{t=1}^T \ddot{z}_{itk}}. \end{aligned}$$
- Model EEE [$\varvec{\Sigma }_k=\lambda \varvec{\Gamma }\varvec{\Delta }\varvec{\Gamma }'$]. In this setting, given that $\varvec{\Sigma }_1=\cdots =\varvec{\Sigma }_K\equiv \varvec{\Sigma }$, maximizing Eq. (3) reduces to the maximization of
  $$\begin{aligned} -\frac{RTI}{2}\log |\varvec{\Sigma }|-\frac{1}{2}\,\text{ tr }(\varvec{\Sigma }^{-1}{\ddot{{\mathbf {Y}}}}). \end{aligned}$$
  Applying Theorem A.2 of Celeux and Govaert (1995), we can update $\varvec{\Sigma }$ as
  $$\begin{aligned} \ddot{\varvec{\Sigma }}= \frac{{\ddot{{\mathbf {Y}}}}}{RTI}. \end{aligned}$$
- Model VEE [$\varvec{\Sigma }_k=\lambda _{k}\varvec{\Gamma }\varvec{\Delta }\varvec{\Gamma }'$]. In this case, it is convenient to write $\varvec{\Sigma }_k=\lambda _{k}{\mathbf {C}}$, where ${\mathbf {C}}= \varvec{\Gamma }\varvec{\Delta }\varvec{\Gamma }'$. Thus, maximizing Eq. (3) reduces to the maximization of
  $$\begin{aligned} -\frac{PR}{2}\sum \limits _{k=1}^{K} \log \lambda _k \sum \limits _{i=1}^{I}\sum \limits _{t=1}^{T}\ddot{z}_{itk}-\sum \limits _{k=1}^{K}\frac{1}{2\lambda _k}\,\text{ tr }\left( {\mathbf {C}}^{-1}{\ddot{{\mathbf {Y}}}}_k\right) . \end{aligned}$$
  Applying Theorem A.1 of Celeux and Govaert (1995), we can update ${\mathbf {C}}$ and $\lambda _{k}$ as
  $$\begin{aligned} {\ddot{{\mathbf {C}}}} = \frac{\sum \limits _{k=1}^K {{\dot{\lambda }}}_k^{-1}{\ddot{{\mathbf {Y}}}}_k}{\left| \sum \limits _{k=1}^K {{\dot{\lambda }}}_k^{-1}{\ddot{{\mathbf {Y}}}}_k\right| ^{\frac{1}{P}}}\quad \text {and} \quad {\ddot{\lambda }}_k = \frac{\,\text{ tr }\left\{ {\ddot{{\mathbf {C}}}}^{-1} {\ddot{{\mathbf {Y}}}}_k \right\} }{PR\sum _{i=1}^I \sum _{t=1}^T \ddot{z}_{itk}}. \end{aligned}$$
- Model EVE [$\varvec{\Sigma }_k=\lambda \varvec{\Gamma }\varvec{\Delta }_{k}\varvec{\Gamma }'$]. Here, maximizing Eq. (3) reduces to the maximization of
  $$\begin{aligned} -\frac{PRTI}{2}\log \lambda -\frac{1}{2\lambda }\sum \limits _{k=1}^{K} \,\text{ tr }(\varvec{\Gamma }'{\ddot{{\mathbf {Y}}}}_k\varvec{\Gamma }\varvec{\Delta }_{k}^{-1}). \end{aligned}$$
  Given that there is no analytical solution for $\varvec{\Gamma }$, while keeping fixed the other parameters, an iterative Minorization-Maximization (MM) algorithm (Browne and McNicholas 2014) is employed. In detail, a surrogate function can be constructed as
  $$\begin{aligned} f\left( \varvec{\Gamma }\right) = \sum \limits _{k=1}^K \,\text{ tr }\left\{ {\ddot{{\mathbf {Y}}}}_k\varvec{\Gamma }\varvec{\Delta }_{k}^{-1}\varvec{\Gamma }'\right\} \le S + \,\text{ tr }\left\{ {{\dot{{\varvec{F}}}}}\varvec{\Gamma }\right\} , \end{aligned}$$
  where *S* is a constant and ${{\dot{{\varvec{F}}}}} = \sum _{k=1}^K\Big (\varvec{\Delta }_{k}^{-1} {{\dot{\varvec{\Gamma }}}}' {\ddot{{\mathbf {Y}}}}_k - e_k \varvec{\Delta }_{k}^{-1} {{\dot{\varvec{\Gamma }}}}'\Big )$, with $e_k$ being the largest eigenvalue of ${\ddot{{\mathbf {Y}}}}_k$. The update of $\varvec{\Gamma }$ is given by ${\ddot{\varvec{\Gamma }}} = {{\dot{{\varvec{G}}}}} {{\dot{{\varvec{H}}}}} '$, where ${{\dot{{\varvec{G}}}}}$ and $\dot{\varvec{H}}$ are obtained from the singular value decomposition of ${{\dot{{\varvec{F}}}}}$. This process is repeated until a specified convergence criterion is met and the update ${\ddot{\varvec{\Gamma }}}$ is obtained. Then, we obtain the update for $\varvec{\Delta }_{k}$ and $\lambda$ as
  $$\begin{aligned} {\ddot{\varvec{\Delta }}}_k = \frac{\text {diag}\left( {\ddot{\varvec{\Gamma }}}' {\ddot{{\mathbf {Y}}}}_k {\ddot{\varvec{\Gamma }}}\right) }{\left| \text {diag}\left( {\ddot{\varvec{\Gamma }}}' {\ddot{{\mathbf {Y}}}}_k {\ddot{\varvec{\Gamma }}}\right) \right| ^\frac{1}{P}}\quad \text {and} \quad {\ddot{\lambda }} = \frac{\sum \limits _{k=1}^K \,\text{ tr }\left( {\ddot{\varvec{\Gamma }}} {\ddot{\varvec{\Delta }}}_k^{-1} {\ddot{\varvec{\Gamma }}}'{\ddot{{\mathbf {Y}}}}_k\right) }{PRTI}. \end{aligned}$$
- Model VVE [$\varvec{\Sigma }_k=\lambda _{k}\varvec{\Gamma }\varvec{\Delta }_{k}\varvec{\Gamma }'$]. In this case, maximizing Eq. (3) reduces to the maximization of
  $$\begin{aligned} -\frac{PR}{2}\sum \limits _{k=1}^{K} \log \lambda _k \sum \limits _{i=1}^{I}\sum \limits _{t=1}^{T}\ddot{z}_{itk}-\sum \limits _{k=1}^{K}\frac{1}{2\lambda _k}\,\text{ tr }(\varvec{\Gamma }'{\ddot{{\mathbf {Y}}}}_k\varvec{\Gamma }\varvec{\Delta }_{k}^{-1}). \end{aligned}$$
  Again, there is no analytical solution for $\varvec{\Gamma }$, and its update is obtained by employing the MM algorithm as described for the EVE model. Then, the updates for $\varvec{\Delta }_{k}$ and $\lambda _{k}$ are
  $$\begin{aligned} {\ddot{\varvec{\Delta }}}_k = \frac{\text {diag}\left( {\ddot{\varvec{\Gamma }}}' {\ddot{{\mathbf {Y}}}}_k {\ddot{\varvec{\Gamma }}}\right) }{\left| \text {diag}\left( {\ddot{\varvec{\Gamma }}}' {\ddot{{\mathbf {Y}}}}_k {\ddot{\varvec{\Gamma }}}\right) \right| ^\frac{1}{P}}\quad \text {and} \quad {\ddot{\lambda }}_k = \frac{\left| \text {diag}\left( {\ddot{\varvec{\Gamma }}}' {\ddot{{\mathbf {Y}}}}_k {\ddot{\varvec{\Gamma }}}\right) \right| ^{\frac{1}{P}}}{R\sum _{i=1}^I \sum _{t=1}^T \ddot{z}_{itk}}. \end{aligned}$$
- Model EEV [$\varvec{\Sigma }_k=\lambda \varvec{\Gamma }_{k}\varvec{\Delta }\varvec{\Gamma }_{k}'$]. Here, maximizing Eq. (3) reduces to the maximization of
  $$\begin{aligned} -\frac{PRTI}{2}\log \lambda -\frac{1}{2\lambda }\sum \limits _{k=1}^{K} \,\text{ tr }(\varvec{\Gamma }_{k}'{\ddot{{\mathbf {Y}}}}_k\varvec{\Gamma }_{k}\varvec{\Delta }^{-1}). \end{aligned}$$
  An algorithm similar to the one proposed by Celeux and Govaert (1995) can be employed here. In detail, the eigen-decomposition ${\mathbf {Y}}_k={\varvec{L}}_k \varvec{\Omega }_k {\varvec{L}}_k'$ is firstly considered, with the eigenvalues in the diagonal matrix $\varvec{\Omega }_k$ following descending order and orthogonal matrix ${\varvec{L}}_k$ composed of the corresponding eigenvectors. Then, we obtain the update for $\varvec{\Gamma }_k$, $\varvec{\Delta }$ and $\lambda$ as
  $$\begin{aligned} {\ddot{\varvec{\Gamma }}}_k={\ddot{{\varvec{L}}}}_k , \quad {\ddot{\varvec{\Delta }}} = \frac{\sum \limits _{k=1}^K {\ddot{\varvec{\Omega }}}_k}{\left| \sum \limits _{k=1}^K {\ddot{\varvec{\Omega }}}_k\right| ^\frac{1}{P}}\quad \text {and} \quad {\ddot{\lambda }} = \frac{\left| \sum \limits _{k=1}^K {\ddot{\varvec{\Omega }}}_k\right| ^\frac{1}{P}}{RTI}. \end{aligned}$$
- Model VEV [$\varvec{\Sigma }_k=\lambda _k\varvec{\Gamma }_{k}\varvec{\Delta }\varvec{\Gamma }_{k}'$]. In this setting, maximizing Eq. (3) reduces to the maximization of
  $$\begin{aligned} -\frac{PR}{2}\sum \limits _{k=1}^{K} \log \lambda _k \sum \limits _{i=1}^{I}\sum \limits _{t=1}^{T}{\ddot{z}}_{itk}-\sum \limits _{k=1}^{K}\frac{1}{2\lambda _k}\,\text{ tr }(\varvec{\Gamma }_k'{\ddot{{\mathbf {Y}}}}_k\varvec{\Gamma }_k\varvec{\Delta }^{-1}). \end{aligned}$$
  By using the same algorithm applied for the EEV model, the updates for $\varvec{\Gamma }_k$, $\varvec{\Delta }_{k}$ and $\lambda _{k}$ are
  $$\begin{aligned} {\ddot{\varvec{\Gamma }}}_k= & {} {\ddot{{\varvec{L}}}}_k , \quad {\ddot{\varvec{\Delta }}} = \frac{\sum \limits _{k=1}^K \lambda _k^{-1} {\ddot{\varvec{\Omega }}}_k}{\left| \sum \limits _{k=1}^K \lambda _k^{-1} {\ddot{\varvec{\Omega }}}_k\right| ^\frac{1}{P}}\quad \text {and} \\ {\ddot{\lambda }}_k= & {} \frac{\,\text{ tr }\left\{ {\ddot{\varvec{\Omega }}}_k {\ddot{\varvec{\Delta }}}^{-1} \right\} }{PR\sum _{i=1}^I \sum _{t=1}^T \ddot{z}_{itk}}. \end{aligned}$$
- Model EVV [$\varvec{\Sigma }_k=\lambda \varvec{\Gamma }_{k}\varvec{\Delta }_{k}\varvec{\Gamma }_{k}'$]. For this model, we firstly write ${\mathbf {C}}_k = \varvec{\Gamma }_k\varvec{\Delta }_k\varvec{\Gamma }_k'$. Then, maximizing Eq. (3) reduces to the maximization of
  $$\begin{aligned} -\frac{PRTI}{2}\log \lambda -\frac{1}{2\lambda }\sum \limits _{k=1}^{K} \,\text{ tr }({\ddot{{\mathbf {Y}}}}_k{\mathbf {C}}_k^{-1}). \end{aligned}$$
  The updates of this model can be obtained in a similar fashion of the EVI model, except for the fact that ${\mathbf {C}}_k$ is not diagonal. Thus, by employing Theorem A.1 of Celeux and Govaert (1995) we can update ${\mathbf {C}}_k$ and $\lambda$ as
  $$\begin{aligned} {\ddot{{\mathbf {C}}}}_k = \frac{{\ddot{{\mathbf {Y}}}}_k}{\left| {\ddot{{\mathbf {Y}}}}_k\right| ^{\frac{1}{P}}}\quad \text {and} \quad {\ddot{\lambda }} = \frac{\sum \limits _{k=1}^K \left| {\ddot{{\mathbf {Y}}}}_k\right| ^{\frac{1}{P}}}{RTI}. \end{aligned}$$
- Model VVV [$\varvec{\Sigma }_k=\lambda _{k}\varvec{\Gamma }_{k}\varvec{\Delta }_{k}\varvec{\Gamma }_{k}'$]. In the case well-known case, maximizing Eq. (3) reduces to the maximization of
  $$\begin{aligned} -\frac{R}{2}\sum \limits _{k=1}^K\log |\varvec{\Sigma }_k|\sum \limits _{i=1}^{I}\sum \limits _{t=1}^{T}{\ddot{z}}_{itk}-\frac{1}{2}\sum \limits _{k=1}^K\,\text{ tr }\left( \varvec{\Sigma }_k^{-1}{\ddot{{\mathbf {Y}}}}_k\right) . \end{aligned}$$
  Applying Theorem A.2 of Celeux and Govaert (1995), we update $\varvec{\Sigma }_k$ as
  $$\begin{aligned} {\ddot{\varvec{\Sigma }}}_k = \frac{{\ddot{{\mathbf {Y}}}}_k}{R\sum _{i=1}^I \sum _{t=1}^T \ddot{z}_{itk}}. \end{aligned}$$

*CM-Step 2* At the second CM-step, we maximize the expectation of the complete-data log-likelihood with respect to $\varvec{\Theta }_{2}$, keeping $\varvec{\Theta }_{1}$ fixed at ${\ddot{\varvec{\Theta }}}_{1}$. The update for $\varvec{\Psi }_k$ depends on which of the 7 parsimonious structures is considered. For notational simplicity, let ${\ddot{{\mathbf {W}}}}=\sum _{k=1}^K {\ddot{{\mathbf {W}}}}_k$ be the update of the within state column scatter matrix, where ${\ddot{{\mathbf {W}}}}_k = \sum _{i=1}^I \sum _{t=1}^T \ddot{z}_{itk}\left( {\mathbf {X}}_{it}-{\ddot{{\mathbf {M}}}}_k\right) '{\ddot{\varvec{\Sigma }}}_k^{-1}\left( {\mathbf {X}}_{it}-{\ddot{{\mathbf {M}}}}_k\right)$ is the update of the column scatter matrix related to the *k*th state. In detail, we have:

- Model II [$\varvec{\Psi }_k={\varvec{I}}$]. This is the simplest model and no parameters need to be estimated.
- Model EI [$\varvec{\Psi }_k=\varvec{\Delta }$]. In this setting, maximizing Eq. (3) reduces to the maximization of
  $$\begin{aligned} -\frac{1}{2}\,\text{ tr }\left( {\ddot{{\mathbf {W}}}}\varvec{\Delta }^{-1}\right) . \end{aligned}$$
  Applying Corollary A.1 of Celeux and Govaert (1995), we can obtain $\varvec{\Delta }$ as
  $$\begin{aligned} {\ddot{\varvec{\Delta }}} = \frac{\text {diag}\left( {\ddot{{\mathbf {W}}}}\right) }{\left| \text {diag}\left( {\ddot{{\mathbf {W}}}}\right) \right| ^\frac{1}{R}}. \end{aligned}$$
- Model VI [$\varvec{\Psi }_k=\varvec{\Delta }_k$]. Here, maximizing Eq. (3) reduces to the maximization of
  $$\begin{aligned} -\frac{1}{2}\sum \limits _{k=1}^K\,\text{ tr }\left( {\ddot{{\mathbf {W}}}}_k\varvec{\Delta }_k^{-1}\right) . \end{aligned}$$
  Applying Corollary A.1 of Celeux and Govaert (1995), we can update $\varvec{\Delta }_k$ as
  $$\begin{aligned} {\ddot{\varvec{\Delta }}}_k = \frac{\text {diag}\left( {\ddot{{\mathbf {W}}}}_k\right) }{\left| \text {diag}\left( {\ddot{{\mathbf {W}}}}_k\right) \right| ^\frac{1}{R}}. \end{aligned}$$
- Model EE [$\varvec{\Psi }_k=\varvec{\Gamma }\varvec{\Delta }\varvec{\Gamma }'$]. In this case, given that $\varvec{\Psi }_1=\cdots =\varvec{\Psi }_K\equiv \varvec{\Psi }$, maximizing Eq. (3) reduces to the maximization of
  $$\begin{aligned} -\frac{1}{2}\,\text{ tr }\left( {\ddot{{\mathbf {W}}}}\varvec{\Psi }^{-1}\right) . \end{aligned}$$
  Applying Theorem A.2 of Celeux and Govaert (1995), we can update $\varvec{\Psi }$ as
  $$\begin{aligned} {\ddot{\varvec{\Psi }}} = \frac{{\ddot{{\mathbf {W}}}}}{\left| {\ddot{{\mathbf {W}}}} \right| ^\frac{1}{R}}. \end{aligned}$$
- Model VE [$\varvec{\Psi }_k=\varvec{\Gamma }\varvec{\Delta }_k\varvec{\Gamma }'$]. In this setting, maximizing Eq. (3) reduces to the maximization of
  $$\begin{aligned} -\frac{1}{2}\sum \limits _{k=1}^K\,\text{ tr }\left( \varvec{\Gamma }'{\ddot{{\mathbf {W}}}}_k\varvec{\Gamma }\varvec{\Delta }_k^{-1}\right) . \end{aligned}$$
  Similarly to the EVE and VVE models in the CM-Step 1, there is no analytical solution for $\varvec{\Gamma }$, while keeping fixed the other parameters. Therefore, the MM algorithm is implemented by following the same procedure explained for the EVE model and by replacing ${\ddot{{\mathbf {Y}}}}$ with ${\ddot{{\mathbf {W}}}}$. Then, the update of $\varvec{\Delta }_k$ is
  $$\begin{aligned} {\ddot{\varvec{\Delta }}}_k= \frac{\text {diag}\left( {\ddot{\varvec{\Gamma }}}' {\ddot{{\mathbf {W}}}}_k {\ddot{\varvec{\Gamma }}}\right) }{\left| \text {diag}\left( {\ddot{\varvec{\Gamma }}}' {\ddot{{\mathbf {W}}}}_k {\ddot{\varvec{\Gamma }}}\right) \right| ^\frac{1}{R}}. \end{aligned}$$
- Model EV [$\varvec{\Psi }_k=\varvec{\Gamma }_k\varvec{\Delta }\varvec{\Gamma }_k'$]. Here, maximizing Eq. (3) reduces to the maximization of
  $$\begin{aligned} -\frac{1}{2}\sum \limits _{k=1}^K\,\text{ tr }\left( \varvec{\Gamma }_k'{\ddot{{\mathbf {W}}}}_k\varvec{\Gamma }_k\varvec{\Delta }^{-1}\right) . \end{aligned}$$
  By using the same approach of the EEV and VEV models, and by changing ${\ddot{{\mathbf {Y}}}}$ with ${\ddot{{\mathbf {W}}}}$, we obtain the updates of $\varvec{\Gamma }_k$ and $\varvec{\Delta }$ as
  $$\begin{aligned} {\ddot{\varvec{\Gamma }}}_k={\ddot{{\varvec{L}}}}_k \quad \text {and} \quad {\ddot{\varvec{\Delta }}} = \frac{\sum \limits _{k=1}^K {\ddot{\varvec{\Omega }}}_k}{\left| \sum \limits _{k=1}^K {\ddot{\varvec{\Omega }}}_k\right| ^\frac{1}{R}}. \end{aligned}$$
- Model VV [$\varvec{\Psi }_k=\varvec{\Gamma }_k\varvec{\Delta }_k\varvec{\Gamma }_k'$]. In the full unconstrained case, maximizing Eq. (3) reduces to the maximization of
  $$\begin{aligned} -\frac{1}{2}\sum \limits _{k=1}^K\left( {\ddot{{\mathbf {W}}}}_k\varvec{\Psi }_k^{-1}\right) . \end{aligned}$$
  Applying Theorem A.2 of Celeux and Govaert (1995), we update $\varvec{\Psi }_k$ as
  $$\begin{aligned} {\ddot{\varvec{\Psi }}}_k = \frac{{\ddot{{\mathbf {W}}}}_k}{\left| {\ddot{{\mathbf {W}}}}_k\right| ^\frac{1}{R}}. \end{aligned}$$

**Table 2:** Average MSEs of the parameter estimates for the EII-II MV-HMM. The average is computed among the MSEs of the elements of each estimated parameter, over the *K* states and 50 data sets in each scenario

Dimension	*K*	Parameter	$O_1$		$O_2$	
			$T_1$	$T_2$	$T_1$	$T_2$
$D_1$	2	${\mathbf {M}}$	0.0083	0.0040	0.0063	0.0033
		$\varvec{\Sigma }$	0.0020	0.0016	0.0016	0.0012
		$\varvec{\pi }$	0.0026	0.0029	0.0024	0.0031
		$\varvec{\Pi }$	0.0013	0.0004	0.0010	0.0005
	4	${\mathbf {M}}$	0.0164	0.0084	0.0135	0.0069
		$\varvec{\Sigma }$	0.0029	0.0010	0.0024	0.0013
		$\varvec{\pi }$	0.0022	0.0017	0.0017	0.0021
		$\varvec{\Pi }$	0.0009	0.0006	0.0009	0.0004
$D_2$	2	${\mathbf {M}}$	0.0083	0.0042	0.0064	0.0031
		$\varvec{\Sigma }$	0.0003	0.0002	0.0003	0.0001
		$\varvec{\pi }$	0.0045	0.0042	0.0029	0.0029
		$\varvec{\Pi }$	0.0018	0.0011	0.0012	0.0005
	4	${\mathbf {M}}$	0.0131	0.0071	0.0130	0.0067
		$\varvec{\Sigma }$	0.0004	0.0003	0.0004	0.0001
		$\varvec{\pi }$	0.0017	0.0023	0.0019	0.0021
		$\varvec{\Pi }$	0.0009	0.0005	0.0009	0.0004

#### A note on the initialization strategy

To start our ECM algorithm, we followed the approach of Tomarchio et al. (2020), where a generalization of the short-EM initialization strategy proposed by Biernacki et al. (2003) has been implemented. It consists in *H* short runs of the algorithm from several random positions. The term “short” means that the algorithm is run for a few iterations *s*, without waiting for convergence. In this manuscript, we set $H=100$ and $s=1$. Then, the parameter set producing the largest log-likelihood is used to initialize the ECM algorithm. In both simulated and real data analyses this procedure has shown stable results after multiple runs. Operationally, this initialization strategy is implemented in the HMM.init() function of the **FourWayHMM** package.

## Simulated analyses

### Overview

In this section, we examine different aspects of our MV-HMMs through large-scale simulation studies. Given the high number of models introduced, we will only focus on two of them for the sake of simplicity. In detail, we consider the EII-II MV-HMM, which provides an example of model having the same covariance structure for $\varvec{\Sigma }$ and $\varvec{\Psi }$, and the VVE-EV MV-HMM, which provides an example of model having an opposite covariance structure for $\varvec{\Sigma }$ and $\varvec{\Psi }$. Furthermore, the EII-II MV-HMM is the also the most parsimonious model, whereas the VVE-EV MV-HMM is one of the models for which the MM algorithm is used. For each model, several experimental conditions are evaluated. Specifically, we set $I=100$ and consider two dimensions for the matrices (labeled as $D_1$ when $P=R=2$ and $D_2$ when $P=4$ and $R=8$), two times ($T_1=5$ and $T_2=10$), two number of hidden states ($K=2$ and $K=4$), and two levels of overlap (labeled as $O_1$ and $O_2$). Therefore, $2 \times 2 \times 2 \times 2 = 16$ scenarios are analyzed and, for each of them, 50 data sets are generated by the considered MV-HMM. The parameters used to generate the data are reported in Appendix A.

### Discussion

First of all, we evaluate the recovery and the consistency of the estimated parameters by computing the mean square errors (MSEs). Considering the high number of parameters that should be reported, we follow an approach similar to the one used by Farcomeni and Punzo (2020), i.e. we calculate the average among the MSEs of the elements of each estimated parameter over the *K* states, allowing us to summarize in a single number the MSE of each parameter. Furthermore, before showing the obtained results, it is important to underline the well-known label switching issue, caused by the invariance of the likelihood function under relabeling of the model states (Frühwirth-Schnatter 2006). There are no generally accepted labeling methods, and we simply attribute the labels by looking at the estimated ${\mathbf {M}}_k$.

**Table 3:** Average MSEs of the parameter estimates for the VVE-EV MV-HMM. The average is computed among the MSEs of the elements of each estimated parameter, over the *K* states and 50 data sets in each scenario

Dimension	*K*	Parameter	$O_1$		$O_2$	
			$T_1$	$T_2$	$T_1$	$T_2$
$D_1$	2	${\mathbf {M}}$	0.0095	0.0042	0.0069	0.0033
		$\varvec{\Sigma }$	0.0083	0.0032	0.0076	0.0039
		$\varvec{\Psi }$	0.0025	0.0014	0.0022	0.0011
		$\varvec{\pi }$	0.0032	0.0026	0.0025	0.0018
		$\varvec{\Pi }$	0.0015	0.0001	0.0007	0.0005
	4	${\mathbf {M}}$	0.0135	0.0073	0.0113	0.0054
		$\varvec{\Sigma }$	0.0101	0.0055	0.0098	0.0050
		$\varvec{\Psi }$	0.0038	0.0018	0.0034	0.0015
		$\varvec{\pi }$	0.0020	0.0018	0.0022	0.0018
		$\varvec{\Pi }$	0.0008	0.0004	0.0008	0.0004
$D_2$	2	${\mathbf {M}}$	0.0072	0.0037	0.0067	0.0035
		$\varvec{\Sigma }$	0.0006	0.0004	0.0007	0.0003
		$\varvec{\Psi }$	0.0032	0.0016	0.0031	0.0016
		$\varvec{\pi }$	0.0034	0.0027	0.0034	0.0022
		$\varvec{\Pi }$	0.0010	0.0006	0.0010	0.0004
	4	${\mathbf {M}}$	0.0720	0.0225	0.0169	0.0095
		$\varvec{\Sigma }$	0.0142	0.0061	0.0018	0.0008
		$\varvec{\Psi }$	0.0342	0.0171	0.0062	0.0033
		$\varvec{\pi }$	0.0068	0.0031	0.0021	0.0019
		$\varvec{\Pi }$	0.0011	0.0007	0.0007	0.0004

Tables 2 and 3 report the average MSEs, computed after fitting the EII-II and VVE-EV MV-HMMs, with the corresponding *K*, to the respective data sets. Note that the column covariance matrix $\varvec{\Psi }$ is not reported in Table 2 since it is not estimated in the EII-II MV-HMM.

As we can see, the MSEs can be considered negligible in all the considered scenarios. Regardless of the data dimensionality, it is interesting to note that, for a fixed overlap, their values become better with the increase of *T*. Note also that, fixed *T*, their values generally improve as we move from $O_1$ to $O_2$, thus confirming the lower separation among the states. Additionally, when the VVE-EV MV-HMM is considered, it seems that the MM algorithm used for estimating $\varvec{\Sigma }_k$ produces reliable values.

**Fig. 1:**
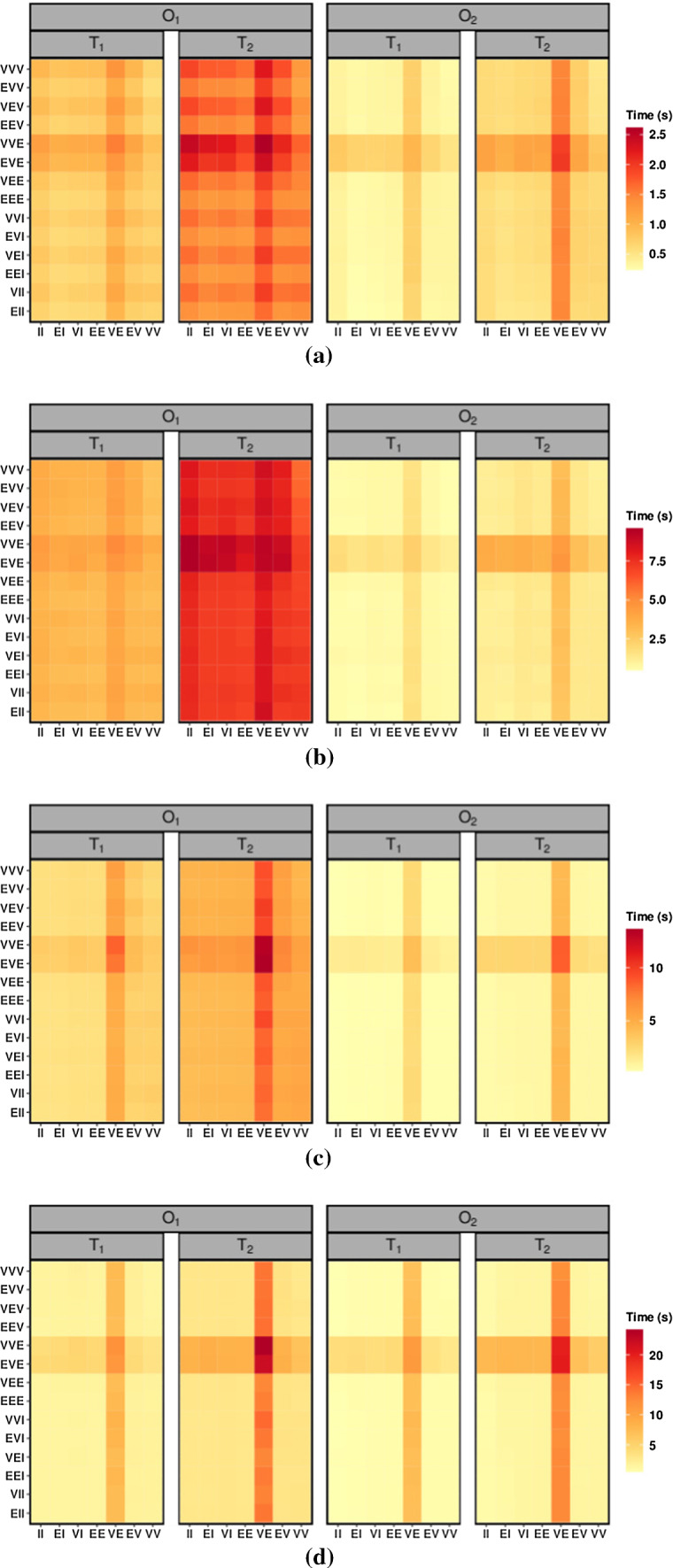
Heat maps of the average computational time for fitting the 98 MV-HMMs, computed over 50 data sets generated by a EII-II MV-HMM with $D_1$ and $K=2$ **(a)**, $D_1$ and $K=4$ **(b)**, $D_2$ and $K=2$ **(c)**, $D_2$ and $K=4$ **(d)**

**Fig. 2:**
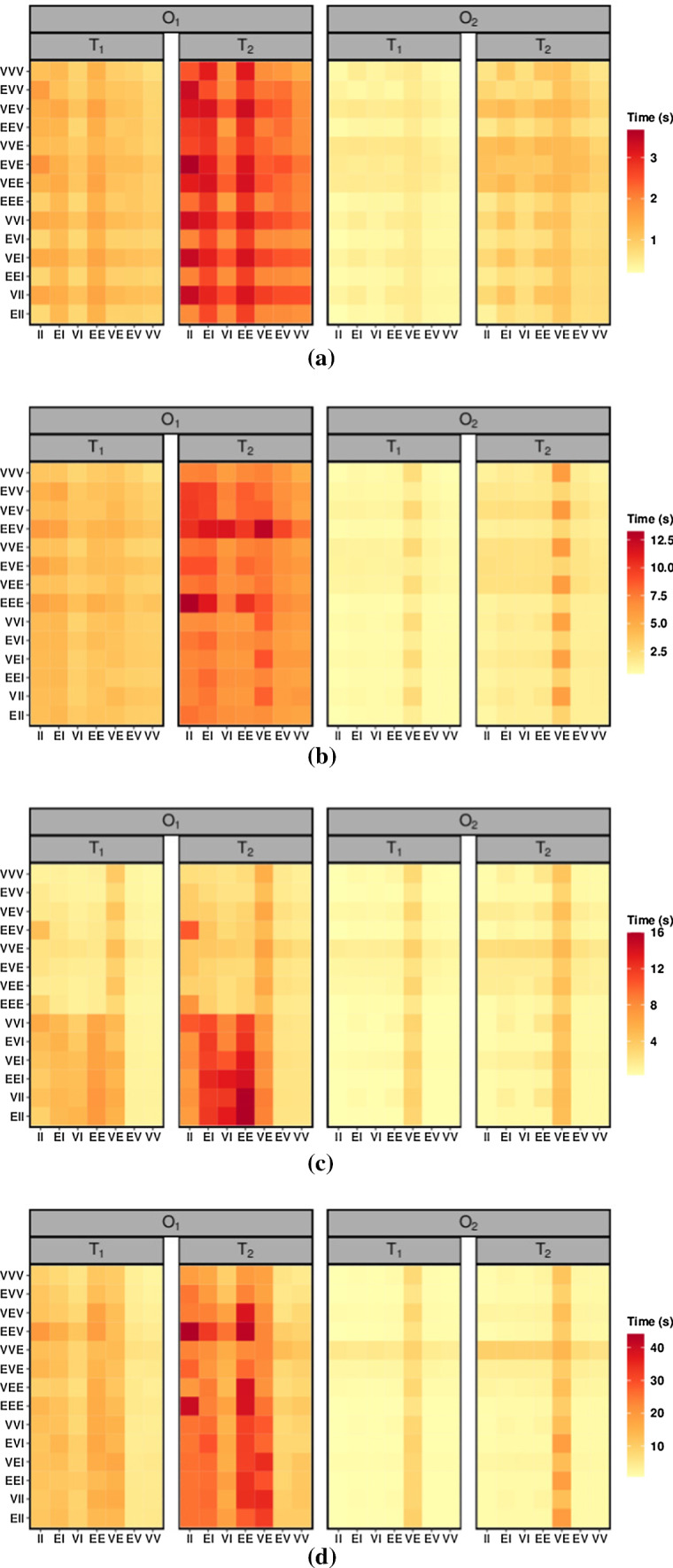
Heat maps of the average computational time for fitting the 98 MV-HMMs, computed over 50 data sets generated by a VVE-EV MV-HMM with $D_1$ and $K=2$ **(a)**, $D_1$ and $K=4$ (b), $D_2$ and $K=2$ **(c)**, $D_2$ and $K=4$ **(d)**

Another aspect that is interesting to evaluate is the computational time required for fitting the MV-HMMs. In detail, on each of the above data sets, all the 98 MV-HMMs are now fitted for the corresponding *K*, and their computational times (in seconds) are illustrated by using the heat maps of Figs. 1 and 2.

Computation is performed on a Windows 10 PC, with AMD Ryzen 7 3700x CPU, 16.0 GB RAM, using the R 64-bit statistical software, and the proc.time() function of the **base** package is used to measure the elapsed time. As it is reasonable to expect, the computational time grows as *T* increases on each scenario, and it decreases when we pass from $O_1$ to $O_2$, highlighting the easier estimation in the latter case. Furthermore, the computational time roughly triplicates when we move from fitting MV-HMMs with $K=2$ to MV-HMMs with $K=4$ hidden states, and approximately quadruplicates when we compare $D_1$ to $D_2$. It is interesting to note that the EVE-VE and VVE-VE MV-HMMs, which are the two models for which we use a MM algorithm for estimating both covariance matrices, are the most time consuming, with a computational burden that seems to double with respect to the other models. This is particularly evident in the $O_2$ scenarios.

The total computational time can be strongly reduced by exploiting parallel computing. In detail, Table 4 shows the overall time taken by fitting the 98 MV-HMMs sequentially (default in R) and by parallelizing them on 14 cores. As we can see, the computational burden is decreased by about 10 times, and all the models can be fitted in a reasonable fast way (with some exceptions in the $O_1$ scenarios).

**Table 4:** Average computational times (in seconds) for fitting all the 98 HHMs with *K* states over the 50 data sets generated by the EII-II MV-HMM and VVE-EV MV-HMM on each scenario

Dimension	MV-HMM	Type	*K*	$O_1$		$O_2$	
				$T_1$	$T_2$	$T_1$	$T_2$
$D_1$	EII-II	Sequential	2	82.38	159.96	41.37	78.03
			4	346.51	753.71	89.32	176.54
		Parallel	2	9.65	15.53	6.64	9.42
			4	29.87	60.01	10.39	17.34
	VVE-EV	Sequential	2	112.23	243.09	39.62	87.13
			4	373.85	730.61	93.08	193.70
		Parallel	2	10.55	20.23	5.21	8.84
			4	31.12	59.62	9.43	17.27
$D_2$	EII-II	Sequential	2	289.68	548.37	75.23	146.05
			4	285.00	531.69	202.20	374.94
		Parallel	2	24.85	44.17	8.37	14.87
			4	25.14	46.56	19.28	33.6
	VVE-EV	Sequential	2	268.88	529.20	85.49	149.50
			4	981.04	2007.42	206.9	378.85
		Parallel	2	22.99	43.48	9.10	13.70
			4	78.09	167.51	20.68	39.08

Lastly, the capability of the Bayesian information criterion (BIC; Schwarz et al. 1978) in identifying the true parsimonious structure and the correct number of groups is investigated. This is because, so far, we have fitted models with *K* equal to the true number of states, and we need to assess if the BIC, which is one of the most famous and used tools in model-based clustering, accurately works. Therefore, on each of the above data sets, the 98 MV-HMMs are fitted for $K\in \left\{ 1,\ldots ,K^*+1\right\}$, where $K^*$ is the true number of states, and the number of times for which the true parsimonious structure is selected by the BIC are reported in Table 5. First of all, in each scenario, the true $K^*$ has been almost always selected by the best fitting model according to the BIC, with only 6 exceptions for the VVE-EV model with dimension $D_2$, overlap $O_1$, $K=4$ states and $T_1$ times, and 3 exceptions for the VVE-EV with dimension $D_2$, overlap $O_1$, $K=4$ states and $T_2$ times. Additionally, we notice that in almost all the cases the parsimonious structure of the true data generating model has been identified by the BIC. In those few cases where the BIC selects a wrong model, this is because of an incorrect choice of the parsimonious structure for one of the two covariance matrices $\varvec{\Sigma }$ or $\varvec{\Psi }$.

**Table 5:** Number of times, over the 50 data sets generated by the two MV-HMMs on each scenario, for which the true parsimonious structure is selected by the BIC when all the 98 MV-HMMs are fitted for $K\in \left\{ 1,\ldots ,K^*+1\right\}$

Dimension	MV-HMM	*K*	$O_1$		$O_2$	
			$T_1$	$T_2$	$T_1$	$T_2$
$D_1$	EII-II	2	47	48	49	49
		4	46	50	50	50
	VVE-EV	2	48	50	50	48
		4	50	50	50	50
$D_2$	EII-II	2	48	49	49	50
		4	50	50	50	50
	VVE-EV	2	50	50	50	50
		4	45	49	50	50

## Real data example

### Overview

In this section, we analyze data concerning the unemployment rate in the Italian provinces (NUTS3, according to the European Nomenclature of Territorial Units for Statistics). The data comes from the Italian National Institute of Statistics (ISTAT), a public research organization and the main producer of official statistics in the service of citizens and policy-makers in Italy, and are freely accessible at http://dati.istat.it/#. In detail, we investigate the $I=98$ Italian provinces for which the unemployment rate is available from the beginning of the data collection at the provincial level (2004) to 2019. This implies that we are considering $T=16$ years of data. Note that, to obtain a balanced dataset, some provinces are not included in the analysis since they were available for only few years.

For each province, the unemployment rate is recorded in a two-factor format. The first factor, gender, has two levels (i.e. $P=2$): males and females. The second factor, age, has three levels (i.e. $R=3$) driven by the age class: 15–24, 25–34 and 35–older. Therefore, the whole data set is presented in a four-way array having dimension $2 \times 3 \times 98 \times 16$.

In analyzing this data set, several aspects are worth to be investigated. The first concerns the existence of areas with similar unemployment levels among the Italian provinces. According to the existing literature on this topic (see, e.g., Cracolici et al. 2007, 2009), unemployment rates appear to vary widely across the country, but when analyzed at provincial level tend to be spatially clustered; in other terms, provinces show a certain amount of spatial autocorrelation. To include such information in the analysis, we implemented to matrix-variate longitudinal data an approach similar to that introduced by Scrucca (2005). Specifically, Scrucca (2005) proposed a clustering procedure based on the standardized Getis and Ord measure of local spatial autocorrelation (Getis and Ord 1992), herein labeled as *G*. He applied such approach for the analysis of the unemployment rates of the municipalities in the Umbria region (NUTS2), but similar implementations have been also done in other applicative fields (see, e.g. Holden and Evans 2010 and Appice et al. 2013). In our case, to implement this approach we

- computed a $I \times I$ symmetric spatial weight matrix which takes values equal to 1 for neighbouring provinces and 0 otherwise. We define neighbours via the symmetric relative graph criterion (Toussaint 1980 and Jaromczyk and Toussaint 1992).
- computed, for a fixed *t*, ${\mathbf {x}}_{it}=\text {vec}({\mathbf {X}}_{it})$, where $\text {vec}(\cdot )$ is the vectorization operator, thus transforming the $P \times R$ matrices of each province into $PR-$dimensional vectors. Then, we calculated $G_j({\mathbf {x}}_{it})$ for the *j*-th variable ($j=1,\ldots ,PR$) on the *i*-th unit ($i=1,\ldots ,I$) as in Scrucca (2005). Such a procedure is repeated for each *t*, with $t=1,\ldots ,T$. From an interpretative point of view, high (low) positive values of $G_j({\mathbf {x}}_{it})$ indicate the possibility of a local cluster of high (low) unemployment rates concerning the *i*-th province and its neighborhood. The obtained values, which contain both spatial and unemployment information, are lastly re-arranged in the original (for each province) $P \times R$ matrix-variate structure and used in the subsequent analyses.

Another aspect of interest is the strength of time dependence as measured by the transition probability matrix, as well as how the provinces move between the hidden states. This latter aspect can be particularly of interest in light of the great recession globally occurred in 2007–2009, and which has led Italy to be one of the most affected countries.

As mentioned in Sect. 1, we compare the performance of our models with those of two alternative approaches that could be used if our models were not available:

1. mixtures of parsimonious matrix-variate normal distributions (MVN-Ms). To use such model, we collapsed the *I* and *T* dimensions into an unique *IT* dimension, obtaining a $P \times R \times IT$ array. In doing this, we are removing the modelization of the temporal structure of the data as well as losing interpretability because of the coercion of the data in a three-way array, leading to the issues discussed at the points (a) and (b) of Sect. 1. A total of 98 parsimonious models is still obtained;
2. parsimonious multivariate normal HMMs (M-HMMs). To use these models, we vectorize the $P \times R$ matrices of each province into $PR-$dimensional vectors, thus obtaining a $PR \times I \times T$ array. Thus, while in this way we are still modeling the temporal structure of the data, the estimated model has the disadvantages mentioned at the point (a) of Sect. 1. Notice that, in this case we have a total of 14 parsimonious models.

### Discussion

All the competing models are fitted to the data for $K\in \left\{ 1,\ldots ,9\right\}$ and the corresponding results are reported in Table 6.

**Table 6:** Parsimonious structure (Pars), number of states (*K*) and value of the information criterion (BIC) for the best among each competing model according to the BIC

Model	Pars	*K*	BIC
**MV-HMMs**	**VEE-EE**	**8**	**13890**.**34**
M-HMMs	VEE	8	13942.92
MVN-Ms	VEE-VE	6	17451.99

Firstly, we notice that the overall best model according to the BIC is the VEE-EE MV-HMM with $K=8$ hidden states. A similar number of states is also detected by the best M-HMMs, having a VEE parsimonious structure but a worse BIC than our best model. Conversely, $K=6$ hidden states are chosen for the best among the MVN-Ms which, despite the similar parsimonious structure to our best model, has by far the worst BIC value. Thus, the obtained results seem to suggest that (i) the modelization of the temporal structure is relevant for our data and (ii) the data coercion leads to worst fitting performance.

By focusing on the VEE-EE MV-HMM, and before graphically showing how the detected states cluster the Italian provinces, useful insights can be gained by looking at its estimated parameters. Specifically, the estimated mean matrices for the hidden states are

$$\begin{aligned} {\mathbf {M}}_1= & {} \begin{bmatrix} -1.63 &{} -1.55 &{} -1.47 \\ -1.48 &{} -1.47 &{} -1.49 \end{bmatrix}, \\ {\mathbf {M}}_2= & {} \begin{bmatrix} -1.06 &{} -1.18 &{} -1.15 \\ -1.04 &{} -1.24 &{} -1.16 \end{bmatrix},\\ {\mathbf {M}}_3= & {} \begin{bmatrix} -0.64 &{} -0.82 &{} -0.75\\ -0.58 &{} -0.80 &{} -0.62 \end{bmatrix}, \\ {\mathbf {M}}_4= & {} \begin{bmatrix} 0.08 &{} -0.04 &{} -0.17 \\ 0.20 &{} -0.08 &{} 0.06 \end{bmatrix},\\ {\mathbf {M}}_5= & {} \begin{bmatrix} 0.77 &{} 0.89 &{} 0.59\\ 0.59 &{} 0.98 &{} 0.60 \end{bmatrix}, \quad {\mathbf {M}}_6= \begin{bmatrix} 1.22 &{} 1.30 &{} 1.48 \\ 1.11 &{} 1.51 &{} 1.48 \end{bmatrix},\\ {\mathbf {M}}_7= & {} \begin{bmatrix} 1.82 &{} 2.25 &{} 1.89 \\ 1.67 &{} 1.93 &{} 1.53 \end{bmatrix}, \quad {\mathbf {M}}_8= \begin{bmatrix} 2.47 &{} 2.71 &{} 3.08 \\ 2.41 &{} 2.91 &{} 2.34 \end{bmatrix}. \end{aligned}$$

As we can note, it is possible to sort the states according to growing unemployment levels, both in the gender and ages factors. More in detail, as we move from the first to the eighth state the unemployment levels rise, and each state becomes worse than the previous ones under any point of view. We can also observe that in the first four states the unemployment levels for males are lower or very similar than those of females, whereas in the last four states an opposite behavior seems to occur. It might be also interesting to report that in the first and the fifth states the differences between the two genders decrease as the age classes increase, whereas in the seventh and eighth states (i.e. the worst states) such differences become larger for growing age classes.

As for the gender-related covariance structure, we have different volumes (${\widehat{\lambda }}_1=0.11$, ${\widehat{\lambda }}_2=0.13$, ${\widehat{\lambda }}_3=0.19$, ${\widehat{\lambda }}_4=0.32$, ${\widehat{\lambda }}_5=0.37$, ${\widehat{\lambda }}_6=0.30$, ${\widehat{\lambda }}_7=0.33$ and ${\widehat{\lambda }}_8=0.53$) but the following common orientation and shape matrices

$$\begin{aligned} {\widehat{\varvec{\Delta }}}=\begin{bmatrix} 1.16 &{} 0.00 \\ 0.00 &{} 0.86 \end{bmatrix} \quad \text {and} \quad {\widehat{\varvec{\Gamma }}}=\begin{bmatrix} 0.42 &{} -0.91 \\ 0.91 &{} 0.42 \end{bmatrix}. \end{aligned}$$

We can note how the size of the state-scatter, as measured by the volumes, roughly increases as we move from the best to the worst states in terms of unemployment. Instead, there is no need to make the model over-parametrized in terms of shape and orientation because the states share the same PC-orientation ($\varvec{\Gamma }$) along with the normalized variances of these PCs (diagonal elements of $\varvec{\Delta }$); refer to Sect. 2.2. When these quantities are put together to form the state-dependent covariance matrices, we obtain

$$\begin{aligned} \varvec{\Sigma }_1= & {} \begin{bmatrix} 0.10 &{} 0.01 \\ 0.01 &{} 0.12 \end{bmatrix}, \varvec{\Sigma }_2= \begin{bmatrix} 0.11 &{} 0.01 \\ 0.01 &{} 0.14 \end{bmatrix}, \\ \varvec{\Sigma }_3= & {} \begin{bmatrix} 0.17 &{} 0.02 \\ 0.02 &{} 0.21 \end{bmatrix}, \varvec{\Sigma }_4= \begin{bmatrix} 0.29 &{} 0.04 \\ 0.04 &{} 0.35 \end{bmatrix},\\ \varvec{\Sigma }_5= & {} \begin{bmatrix} 0.34 &{} 0.04 \\ 0.04 &{} 0.41 \end{bmatrix}, \varvec{\Sigma }_6= \begin{bmatrix} 0.28 &{} 0.04 \\ 0.04 &{} 0.34 \end{bmatrix}, \\ \varvec{\Sigma }_7= & {} \begin{bmatrix} 0.30 &{} 0.04 \\ 0.04 &{} 0.36 \end{bmatrix}, \varvec{\Sigma }_8= \begin{bmatrix} 0.49 &{} 0.06 \\ 0.06 &{} 0.59 \end{bmatrix}. \end{aligned}$$

We notice that, as we move from the first to the fifth states the variances for both men and women grow. Additionally, the last state has the largest variances for both genders.

As for the estimated age-based covariance matrices

$$\begin{aligned} \varvec{\Psi }_1,\ldots ,\varvec{\Psi }_8= \begin{bmatrix} 1.57 &{} 0.12 &{} 0.15 \\ 0.12 &{} 0.77 &{} 0.10 \\ 0.15 &{} 0.10 &{} 0.87 \end{bmatrix}, \end{aligned}$$

we can note that the variance is higher for the 15–24 age class, and it is relatively similar between the other two age classes.

Lastly, it is worth analyzing the estimated transition probability matrix

$$\begin{aligned} \varvec{\Pi }= \begin{bmatrix} 0.97 &{} 0.03 &{} 0.00 &{} 0.00 &{} 0.00 &{} 0.00 &{} 0.00 &{} 0.00 \\ 0.01 &{} 0.95 &{} 0.04 &{} 0.00 &{} 0.00 &{} 0.00 &{} 0.00 &{} 0.00 \\ 0.00 &{} 0.02 &{} 0.95 &{} 0.03 &{} 0.00 &{} 0.00 &{} 0.00 &{} 0.00 \\ 0.00 &{} 0.00 &{} 0.03 &{} 0.93 &{} 0.04 &{} 0.00 &{} 0.00 &{} 0.00 \\ 0.00 &{} 0.00 &{} 0.00 &{} 0.02 &{} 0.93 &{} 0.05 &{} 0.00 &{} 0.00 \\ 0.00 &{} 0.00 &{} 0.00 &{} 0.00 &{} 0.07 &{} 0.86 &{} 0.07 &{} 0.00 \\ 0.00 &{} 0.00 &{} 0.00 &{} 0.00 &{} 0.00 &{} 0.11 &{} 0.86 &{} 0.03 \\ 0.00 &{} 0.00 &{} 0.00 &{} 0.00 &{} 0.00 &{} 0.00 &{} 0.06 &{} 0.94 \end{bmatrix}. \end{aligned}$$

As we can note by the estimated transition probability matrix, transitions between states mostly occur between adjacent states, whereas they are null among distant states. Furthermore, it seems that the persistence of staying in a state roughly decreases as we move from the first to the seventh. However, it increases for the last state, i.e. it appears more difficult for the provinces clustered in the most troubled state to improve their position.

We have also tested the null hypothesis that the lengths of the segments within each state are geometrically distributed, as assumed by HMMs. To this aim, we defined a simple union-intersection multiple testing procedure based on the intersection of *K* $\chi ^2$ goodness of fit tests, which compare the observed lengths of the segments with the theoretical ones (for other similar tests see, e.g., Maruotti et al. 2021). The obtained (unadjusted) *p*-values are then used to compute the adjusted *p*-values - which are directly comparable with the significance level $\alpha$ - according to the step-down procedure by Holm (1979). We notice that the minimum among the *K* adjusted *p*-values is 0.44; thus, we cannot reject the null hypothesis for any reasonable value of $\alpha$.

**Fig. 3:**
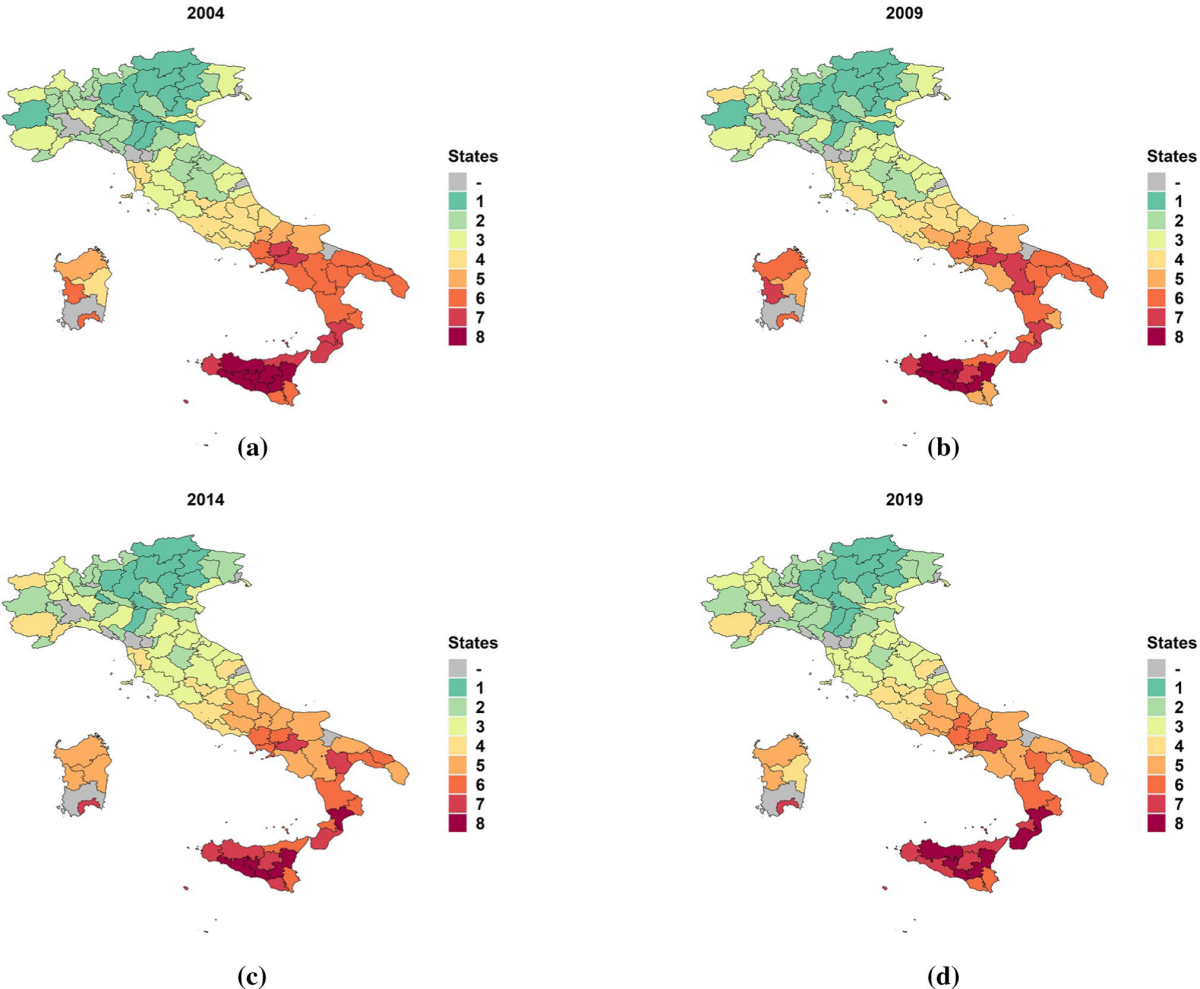
Italian provinces map colored according to the estimated state memberships

We now report information on how the provinces have changed their state over the years and where the detected states are geographically located. This can be better understood by looking at the Italian provinces maps of Fig. 3, that are colored according to state memberships. Note that the provinces not included in the analysis are colored in gray. For simplicity, we avoid to plot a map for each of the 16-years of data, and we limit to report equidistant years covering the entire time period considered.

Starting from the first year of analysis, i.e. 2004, in Figure 3a we can recognize several clusters of provinces that, as we move towards the south, belong to states with higher unemployment rates. After some years characterized by relatively few changes among the states, the economic recession produced its effects in the years 2008–2009, where a lot of provinces, mainly located in the northern central part of the country, started to perform badly (see Figure 3b). In the subsequent years, there has been a certain amount of switches among adjacent states, bringing some provinces to better states and others to worse states (see Figure 3c), despite the overall situation is still relatively distant from that in 2004 for the majority of cases. However, when the last year of analysis is considered in Figure 3d, it is possible to perceive a slight trend change, with signs of recovery especially for the provinces located in the northern central part of the country. In any case, these positive indications are going to be dramatically arrested by the COVID-19 pandemic, and its effects will have serious repercussions in the next years.

## Conclusions

In this manuscript we introduced parsimonious hidden Markov models for matrix-variate balanced longitudinal data. Being (dependent) mixture models, they allow the recovery of homogenous latent subgroups and, simultaneously, provide meaningful interpretation on how the sample units move between the hidden states over time. The parsimony has been introduced via the eigen decomposition of the state covariance matrices, producing a family of 98 MV-HMMs. An ECM algorithm has been illustrated for parameter estimation. At first, the parameter recovery of our algorithm has been evaluated under different scenarios, providing good results. This can be particularly interesting for those MV-HMMs that use a MM algorithm at each step of the ECM algorithm. Relatedly, we have analyzed the computational times for fitting all the 98 MV-HMMs. The computational burden of the MV-HMMs using MM algorithm is definitely higher, even if we are able to fit all the MV-HMMs in a reasonably fast way when parallel computing is considered. The BIC has proven to be reliable in detecting the true number of states in the data as well as the parsimonious structure. The real data example has shown the usefulness of our MV-HMMs. Firstly, when compared with the two alternative approaches and, secondly, in the interpretation of the detected different states at province level.

There are different possibilities for further work, some of which are worth mentioning. First of all, we can extend our MV-HMMs by using skewed or heavy tailed state dependent probability density functions (Gallaugher and McNicholas 2017, 2019; Tomarchio et al. 2020, 2022), in order to model possible features commonly present in the data. A further avenue would deal with the regression setting (Viroli 2012), where covariates shared by all units in the same hidden state are used. This can be done both in a fixed and in random covariates framework (Tomarchio et al. 2021a). Finally, another possibility would be extending our models in order to deal with unbalanced or missing data.

## Parameters used to generate data in Sect. 3

### Scenarios related to $D_1$

As concerns the parameters used to generate the data when $K=2$, we set

- EII-II MV-HHM
  $$\begin{aligned} \varvec{\Sigma }_1= & {} \varvec{\Sigma }_2=1.5{\varvec{I}}\quad \text {and} \\ \varvec{\Psi }_1= & {} \varvec{\Psi }_2={\varvec{I}}, \end{aligned}$$
- VVE-EV MV-HHM
  $$\begin{aligned} \varvec{\Sigma }_1= & {} \begin{bmatrix} 1.43 &{} 0.84 \\ 0.84 &{} 0.88 \end{bmatrix}, \ \varvec{\Sigma }_2= \begin{bmatrix} 2.05 &{} 0.35 \\ 0.35 &{} 1.82 \end{bmatrix}, \\ \varvec{\Psi }_1= & {} \begin{bmatrix} 0.70 &{} 0.33 \\ 0.33 &{} 1.58 \end{bmatrix}, \ \varvec{\Psi }_2= \begin{bmatrix} 1.68 &{} -0.06 \\ -0.06 &{} 0.59 \end{bmatrix}, \end{aligned}$$

while for both MV-HMMs we set $\varvec{\pi }=\left( 0.5,0.5\right)$,

$$\begin{aligned} \varvec{\Pi }= \begin{bmatrix} 0.60 &{} 0.40 \\ 0.20 &{} 0.80 \end{bmatrix}, \quad {\mathbf {M}}_1= \begin{bmatrix} 1.00 &{} 1.50 \\ 0.50 &{} 1.00 \end{bmatrix}. \end{aligned}$$

The mean matrix of the second state (${\mathbf {M}}_2$) is obtained by adding a constant *c* to each element of ${\mathbf {M}}_1$, which depends on the level of overlap. Specifically, we set $c=2$ when $O_1$ is considered, whereas $c=5$ when $O_2$ is examined.

When $K=4$, the first two hidden states have the same $\left\{ \varvec{\Sigma }_k,\varvec{\Psi }_k,{\mathbf {M}}_k; k=1,2\right\}$ as before. Clearly, the covariance matrices of the third and fourth hidden states for the EII-II MV-HMM are still equal to those of the first two states. On the contrary, for the VVE-EV MV-HMM we have

$$\begin{aligned} \varvec{\Sigma }_3= & {} \begin{bmatrix} 0.81 &{} 0.51 \\ 0.51 &{} 0.47 \end{bmatrix}, \ \varvec{\Sigma }_4= \begin{bmatrix} 0.55 &{} 0.20 \\ 0.20 &{} 0.42 \end{bmatrix}, \\ \varvec{\Psi }_3= & {} \begin{bmatrix} 1.45 &{} -0.45 \\ -0.45 &{} 0.82 \end{bmatrix}, \ \varvec{\Psi }_4= \begin{bmatrix} 0.71 &{} -0.34 \\ -0.34 &{} 1.57 \end{bmatrix}. \end{aligned}$$

Then, for both MV-HMMs we set $\varvec{\pi }=\Big (0.25,0.25,0.25,0.25\Big )$ and

$$\begin{aligned} \varvec{\Pi }= \begin{bmatrix} 0.55 &{} 0.00 &{} 0.21 &{} 0.24\\ 0.03 &{} 0.52 &{} 0.18 &{} 0.27\\ 0.06 &{} 0.15 &{} 0.49 &{} 0.30 \\ 0.09 &{} 0.12 &{} 0.33 &{} 0.46 \end{bmatrix}. \end{aligned}$$

To obtain ${\mathbf {M}}_3$ and ${\mathbf {M}}_4$ we add $c=4$ and $c=-2$ to each element of ${\mathbf {M}}_1$, respectively, when $O_1$ is considered. Otherwise, when $O_2$ is considered, we add $c=10$ and $c=-5$ to each element of ${\mathbf {M}}_1$, respectively.

### Scenarios related to $D_2$

As concerns the parameters used to generate the data when $K=2$, we set

- EII-II MV-HMM
  $$\begin{aligned}&\varvec{\Sigma }_1=\varvec{\Sigma }_2=\varvec{\Sigma }_3=\varvec{\Sigma }_4=1.5{\varvec{I}}\quad \text {and}\\&\varvec{\Psi }_1=\varvec{\Psi }_2=\varvec{\Psi }_3=\varvec{\Psi }_4={\varvec{I}}, \end{aligned}$$

- VVE-EV MV-HMM
  $$\begin{aligned} \varvec{\Sigma }_1= & {} \begin{bmatrix} 1.29 &{} -1.16 &{} -0.44 &{} 0.25 \\ -1.16 &{} 1.24 &{} 0.59 &{} -0.33 \\ -0.44 &{} 0.59 &{} 0.69 &{} -0.16 \\ 0.25 &{} -0.33 &{} -0.16 &{} 0.50 \end{bmatrix}, \\ \varvec{\Sigma }_2= & {} \begin{bmatrix} 1.22 &{} -0.61 &{} 0.14 &{} -0.08 \\ -0.61 &{} 0.97 &{} 0.26 &{} -0.13 \\ 0.14 &{} 0.26 &{} 1.36 &{} -0.25 \\ -0.08 &{} -0.13 &{} -0.25 &{} 1.11\end{bmatrix}, \\ \varvec{\Psi }_1= & {} \begin{bmatrix} 0.67&{}0.76&{} -0.01&{}0.72 &{}-0.04&{} -0.60&{} -0.09&{}0.39 \\ 0.76&{}1.27&{}0.65&{}0.67&{}0.07&{} -0.63&{} -0.49&{}0.44 \\ -0.01&{}0.65&{}2.24&{} -0.28&{}0.14&{}0.32&{} -1.16&{} -0.25 \\ 0.72&{}0.67&{} -0.28&{}2.34&{} -0.45&{} -0.56&{}0.00&{}0.44 \\ -0.04&{}0.07&{}0.14&{} -0.45&{}2.05&{}0.41&{} -0.17&{}0.00 \\ -0.60&{} -0.63&{}0.32&{} -0.56&{}0.41&{}1.44&{} -0.18&{}0.04 \\ -0.09&{} -0.49&{} -1.16&{}0.00&{} -0.17&{} -0.18&{}2.15&{} -0.11 \\ 0.39&{}0.44&{} -0.25&{}0.44&{}0.00&{}0.04&{} -0.11&{}1.55 \end{bmatrix}, \\ \varvec{\Psi }_2= & {} \begin{bmatrix} 0.14&{} 0.42&{} -0.01&{} -0.01&{} -0.02&{} -0.06&{} -0.19&{} -0.10 \\ 0.42&{} 2.83 &{} 0.72&{} -0.49&{} -0.07&{} -0.44&{} -0.34&{} -0.66 \\ -0.01&{} 0.72 &{} 1.75&{} -0.04&{} -0.19&{} -0.14&{} 1.05&{} -0.42 \\ -0.01&{} -0.49&{} -0.04&{} 1.58&{} 0.02&{} 0.44&{} -0.47&{} -0.17 \\ -0.02&{} -0.07&{} -0.19&{} 0.02&{} 1.30&{} 0.19&{} 0.59&{} 0.00 \\ -0.06&{} -0.44&{} -0.14&{} 0.44&{} 0.19&{} 1.66&{} -0.03&{} 0.05 \\ -0.19&{} -0.34&{} 1.05&{} -0.47&{} 0.59&{} -0.03&{} 2.78&{} 0.81 \\ -0.10&{} -0.66&{} -0.42&{} -0.17&{} 0.00&{} 0.05&{} 0.81&{} 1.66\end{bmatrix}, \end{aligned}$$

while for both MV-HMMs we set $\varvec{\pi }$ and $\varvec{\Pi }$ as in Sect. A.1, and

$$\begin{aligned} {\mathbf {M}}_1= \begin{bmatrix} 0.51&{} -0.71&{} 0.90&{} -0.84&{} -0.20&{} -0.63&{} 0.09&{} -0.43\\ -1.09&{} -1.71&{} -1.63&{} -1.04&{} -1.38&{} -1.85&{} -1.95&{} -1.76\\ 1.43&{} 1.57&{} 1.34&{} 1.22&{} 1.88&{} 1.50&{} 1.38&{} 1.01\\ 0.64&{} 0.29&{} 0.01&{} 0.03&{} 0.83&{} 0.61&{} 0.09&{} 0.80 \end{bmatrix}. \end{aligned}$$

Similarly to Sect. A.1, we add constants *c* to each element of ${\mathbf {M}}_1$ to obtain ${\mathbf {M}}_2$. In detail, for both models we set $c=0.5$ when $O_1$ is considered, whereas $c=5$ when $O_2$ is examined.

When $K=4$, the first two hidden states have the same $\left\{ \varvec{\Sigma }_k,\varvec{\Psi }_k; k=1,2\right\}$ and ${\mathbf {M}}_1$ as before. Clearly, the covariance matrices of the third and fourth hidden states for the EII-II MV-HMM are still equal to those of the first two states. On the contrary, for the VVE-EV MV-HMM we have

$$\begin{aligned} \varvec{\Sigma }_3= & {} \begin{bmatrix} 1.85 &{} -1.47 &{} -0.60 &{} 0.33 \\ -1.47 &{} 1.80 &{} 0.75 &{} -0.42 \\ -0.60 &{} 0.75 &{} 0.96 &{} -0.34 \\ 0.33 &{} -0.42 &{} -0.34 &{} 0.57 \end{bmatrix}, \\ \varvec{\Sigma }_4= & {} \begin{bmatrix} 1.86 &{} -0.89 &{} 0.16 &{} -0.10 \\ -0.89 &{} 1.52 &{} 0.39 &{} -0.20\\ 0.16 &{} 0.39&{} 1.94 &{}-0.48 \\ -0.10 &{} -0.20 &{}-0.48 &{} 1.44 \end{bmatrix}, \\ \varvec{\Psi }_3= & {} \begin{bmatrix} 0.48&{} 0.06&{} 0.39&{} 0.25&{} -0.21&{} 0.13&{} -0.10&{} 0.36\\ 0.06&{} 0.96&{} 0.45&{} -0.07&{} 0.14&{} 0.37&{} -0.01&{} -0.67\\ 0.39&{} 0.45&{} 1.36&{} 1.06 &{}-0.90&{} -0.05&{} -0.28&{} 0.09\\ 0.25&{} -0.07&{} 1.06&{} 2.31&{} -0.34&{} 0.70&{} -0.38&{} -0.01\\ -0.21&{} 0.14&{} -0.90&{} -0.34&{} 2.07&{} 0.44&{} 0.56&{} -0.28\\ 0.13&{} 0.37&{} -0.05&{} 0.70&{} 0.44&{} 2.75&{} 0.02&{} -0.65\\ -0.10&{} -0.01 &{}-0.28 &{}-0.38&{} 0.56&{} 0.02&{} 1.66&{} -0.29\\ 0.36&{} -0.67&{} 0.09&{} -0.01&{} -0.28&{} -0.65&{} -0.29&{} 2.12 \end{bmatrix},\\ \varvec{\Psi }_4= & {} \begin{bmatrix} 0.52&{} 0.58&{} -0.16 &{} 0.24&{} 0.35&{} 0.18&{} 0.35&{} -0.21\\ 0.58&{} 1.88&{} -0.23&{} 0.77&{} 0.69&{} 0.17&{} 0.12&{} -0.79\\ -0.16&{} -0.23&{} 0.44&{} 0.32&{} 0.37&{} -0.14&{} 0.00&{} 0.27\\ 0.24&{} 0.77&{} 0.32 &{} 1.70&{} 0.43&{} 0.56&{} 0.25&{} -0.23\\ 0.35&{} 0.69&{} 0.37&{} 0.43&{} 1.84&{} 0.22&{} 0.23&{} 0.04\\ 0.18&{} 0.17&{} -0.14&{} 0.56&{} 0.22&{} 2.58&{} 0.60&{} 0.41\\ 0.35&{} 0.12 &{} 0.00&{} 0.25&{} 0.23&{} 0.60&{} 1.90&{} 0.61\\ -0.21&{} -0.79&{} 0.27&{} -0.23 &{}0.04&{} 0.41&{} 0.61&{} 2.85 \end{bmatrix}. \end{aligned}$$

Then, for both MV-HMMs we set $\varvec{\pi }$, $\varvec{\Pi }$ as described in Sect. A.1. Also in this case, we add constants *c* to each element of ${\mathbf {M}}_1$ to obtain the other three mean matrices. Specifically, when $O_1$ is considered, we set $c=1$, $c=-1.5$ and $c=2$ for the EII-II MV-HMM and $c=0.5$, $c=-0.5$ and $c=1$ for the VVE-EV MV-HMM. Conversely, when $O_2$ is considered, we fix $c=5$, $c=10$ and $c=-5$ for both MV-HMMs.
